# Synthesis, Antibacterial Evaluation and Molecular Modeling of Novel Chalcone Derivatives Incorporating the Diphenyl Ether Moiety

**DOI:** 10.3390/molecules30122575

**Published:** 2025-06-13

**Authors:** Shiyuan Li, Hong Jin

**Affiliations:** 1 Key Laboratory of Drug-Targeting and Drug Delivery System, West China School of Pharmacy, Sichuan University, Chengdu 610041, China; li_shiyuan@stu.scu.edu.cn; 2 Key Laboratory of Bio-Resource and Eco-Environment of Ministry of Education, College of Life Sciences, Sichuan University, Chengdu 610065, China

**Keywords:** chalcone, diphenyl ether, antibacterial activity, time-kill, molecular modeling

## Abstract

Twenty-one novel chalcone derivatives, **5a**-**5u**, incorporating a diphenyl ether moiety, were designed, prepared, and subsequently characterized using NMR and HR-MS and FR-IR techniques. Antibacterial evaluation of the target compounds was carried out against *Staphylococcus aureus*, *Escherichia coli*, *Salmonella*, and *Pseudomonas aeruginosa*. The in vitro results demonstrated that most compounds exhibited considerable potency in inhibiting bacterial growth, with MIC values ranging from 25.23 to 83.50 μM for *S. aureus*, 27.53 to 76.25 μM for *E. coli*, 29.73 to 71.73 μM for *Salmonella*, and 27.53 to 71.73 μM for *P. aeruginosa*. Notably, all synthesized compounds exhibited superior antibacterial activity compared to the lead chalcone. In particular, compound **5u**, which features two diphenyl ether moieties, displayed outstanding antibacterial performance, with MIC values of 25.23 μM for *S. aureus* and 33.63 μM for *E. coli*, *Salmonella*, and *P. aeruginosa*. Moreover, compound **5u** outperformed both ciprofloxacin and gentamicin against *Salmonella* and *P. aeruginosa*, and time-kill curve assays further revealed that concentrations of compound **5u** at or above 33.63 μM provided potent and sustained inhibition of both *Salmonella* and *P. aeruginosa*. Additionally, molecular modeling of the *P. aeruginosa* LpxC-compound **5u** complex suggested that compound **5u** could strongly bind to and interact with the binding site of the LpxC. Based on these findings, compound **5u** represents a promising lead for future antimicrobial development.

## 1. Introduction

Bacterial infections have represented a major global health threat for several centuries [1,2,3]. Pathogens such as *Staphylococcus aureus*, *Escherichia coli*, *Salmonella*, and *Pseudomonas aeruginosa* continue to pose significant challenges to healthcare systems [4,5,6]. Alarmingly, antimicrobial resistance (AMR) is projected to cause 10 million deaths annually by 2050 if no effective intervention is implemented [7]. This impending crisis necessitates the development of innovative, broad-spectrum agents capable of overcoming drug resistance. Despite vigorous antibacterial research efforts [8,9,10,11], the current antibiotic pipeline remains critically limited due to protracted development cycles of 10–15 years. The World Health Organization (WHO) has identified AMR as one of the top 10 global health threats, underscoring the urgent need for next-generation therapeutic strategies [12].

**Figure 1:**
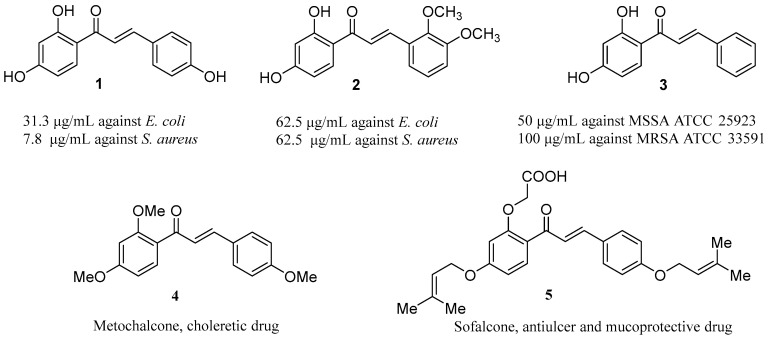
Natural antibacterial chalcones **1**-**3** and chalcone drugs **4**, **5**.

Chalcones, also known as α, β-unsaturated ketones, constitute an important class of natural drug scaffolds [13]. These compounds are widely distributed in vegetables, fruits, teas, and other plants, where they serve as precursors for the biosynthesis of flavonoids and isoflavonoids [14]. The chalcone family has attracted considerable attention due to its diverse biological activities, as exemplified by compounds **1**, **2**, and **3** (Figure 1) [14,15,16,17,18,19]. Several chalcone-based compounds have received clinical approval, including compound **4** (Metochalcone) and compound **5** (Sofalcone) (Figure 1) [20,21]. Numerous reviews have comprehensively discussed chalcone synthesis, biological activities, and molecular targets [13,14,15,16,17,18,19,20,21].

**Figure 2:**
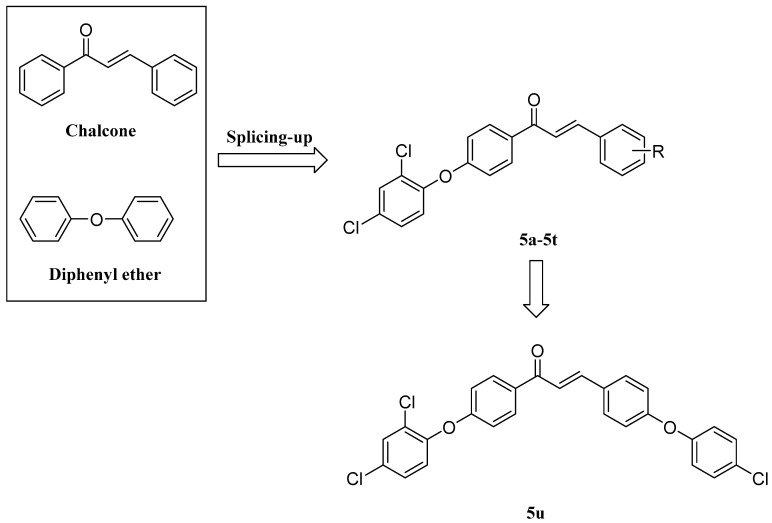
The strategy for design of novel chalcone derivatives **5a**-**5u** with the diphenyl ether moiety.

**Scheme 1:**
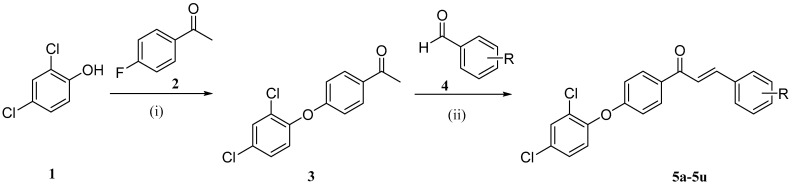
Synthesis of the target compounds. Reagents and conditions: (i) CsCO_3_, 150 °C, 8 h; (ii) NaOH, EtOH, r.t., 5–10 h.

In our previous studies, we synthesized various substituted diphenyl ether derivatives that exhibited significant antimicrobial activity [22,23,24,25]. Natural diphenyl ether derivatives have been reported to possess potent pharmacological activities, including antifungal, antibacterial, antimitotic, and immunosuppressive effects [26,27,28]. To extend our research in developing novel chalcone-based antibacterial agents [29,30,31], we have designed and synthesized a series of new chalcone derivatives, **5a**-**5u**, that incorporate a diphenyl ether moiety, based on the principle of integrating bioactive substructures (Figure 2). Herein, we report the synthesis of these novel chalcone derivatives (Scheme 1), after evaluating their antibacterial activities against *S. aureus*, *E. coli*, *Salmonella*, and *P. aeruginosa*. Furthermore, molecular modeling of the target compound was used to verify their potential mechanism.

## 2. Results and Discussion

### 2.1. Chemistry

The synthetic pathways and general structures of the target compounds **5a**-**5u** are shown in Scheme 1. Initially, compound **3** was obtained via a condensation reaction between compounds **1** and **2** in the presence of CsCO_3_ [32]. Subsequently, compound **3** was reacted with substituted aromatic aldehydes **4** through a classical Aldol condensation, affording a series of novel target compounds **5a**-**5u** [33,34].

Lipinski’s rule of five predicts whether a compound possesses the chemical and physical properties likely to confer oral bioavailability in drugs [35]. In accordance with this principle, we calculated the molecular weight (MW), partition coefficient (logP), number of hydrogen bond acceptors (HBA), and number of hydrogen bond donors (HBD) for all synthesized target compounds (Table 1). Several compounds exhibited parameters consistent with the rule of five, suggesting their potential as orally active drug candidates.

### 2.2. Antibacterial Activity

#### 2.2.1. Minimum Inhibitory Concentration of the Target Compounds **5a**-**5u**

In this study, the target compounds **5a**-**5u** were evaluated for their ability to inhibit bacterial growth using a standard broth minimal inhibitory concentration (MIC) determination (Table 2) [34,36]. The bacterial strains used were *S. aureus*, *E. coli*, *Salmonella*, and *P. aeruginosa*, with ciprofloxacin and gentamicin serving as positive controls. Among the compounds **5a**-**5t**, possessing a single diphenyl ether moiety, most exhibited notable inhibitory effects against the four bacterial species; however, variations in the nature and position of substituents significantly influenced their antibacterial activity. Specifically, our results indicated that both substituent type and position were critical determinants of antibacterial efficacy. For example, compound **5h**, featuring a 3-chloro substituent, demonstrated an MIC of 44.60 µM against *S. aureus*, which is substantially lower than that of its 2-chloro analog, compound **5b** (MIC = 58.65 µM). Additionally, compound **5c**, with a 2-bromo substituent, exhibited broad-spectrum activity against all tested pathogens with a uniform MIC value of 33.48 µM. Notably, compound **5t**, bearing a 4-nitro substituent, showed exceptional inhibition of *P. aeruginosa* (MIC = 32.20 µM), outperforming the analogous compound **5s** with a 3-nitro group (MIC = 40.23 µM).

Furthermore, the compounds demonstrated high efficacy against Gram-negative bacteria, although challenges with *Salmonella* resistance were observed. Several compounds exhibited potent inhibition of Gram-negative bacteria, such as *E. coli*. For example, compound **5c** (2-Br) achieved an MIC value of 33.48 µM against *E. coli*, which is comparable to its activity against *S. aureus*, thereby highlighting its broad-spectrum potential. In contrast, *Salmonella* displayed pronounced resistance; ciprofloxacin, a standard antibiotic, recorded an MIC of 259.17 µM against *Salmonella*, substantially exceeding its efficacy against *E. coli* (MIC = 0.78 µM). Moreover, gentamicin demonstrated a significant increase in MIC against *P. aeruginosa* (MIC = 521.1 µM) relative to *E. coli* (MIC = 1.04 µM), underscoring species-specific sensitivity differences.

In addition, all synthesized compounds exhibited superior antibacterial activity against the four bacterial strains compared to the lead chalcone (Table 2). Incorporation of the diphenyl ether moiety into the chalcone structure substantially enhanced antibacterial properties. Consequently, compound **5u**, which features two diphenyl ether moieties, was synthesized, and it demonstrated the most potent activity, with MIC values of 25.23, 33.63, 33.63, and 33.63 μM against *S. aureus*, *E. coli*, *Salmonella*, and *P. aeruginosa*, respectively. Notably, its activity against *Salmonella* and *P. aeruginosa* surpassed that of ciprofloxacin and gentamicin, thereby warranting further study as a lead compound. As a result, compound **5u** was subjected to in vitro time-kill assays.

#### 2.2.2. Concentration Time-Kill Curves of Compound **5u**

**Figure 3:**
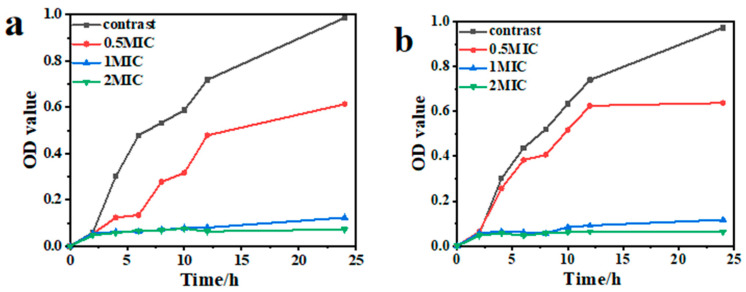
Time-kill analysis of compound **5u** against *Salmonella* (**a**) and *P*. *aeruginosa* (**b**).

Initially, the inhibitory effect of compound **5u** on *Salmonella* was analyzed, revealing significant concentration- and time-dependent characteristics (Figure 3a) [36]. At concentrations equal to or greater than 1 MIC, the antibacterial efficacy was markedly enhanced and sustained. Specifically, after 4 h of treatment, the optical density (OD) values for the 1 MIC and 2 MIC groups decreased to 0.062 and 0.058, respectively, compared to 0.302 for the control, corresponding to approximately 79.5% and 80.8% inhibition. At 24 h, the OD values remained low (1 MIC: 0.124; 2 MIC: 0.073), with the 2 MIC group achieving a 92.6% inhibition rate (control OD at 24 h: 0.987), thereby demonstrating long-term suppression of bacterial growth at higher concentrations. In contrast, the 0.5 MIC concentration exhibited only transient efficacy with rebound tendencies, while the OD value increased from 0.135 at 6 h to 0.278 at 8 h, and further to 0.614 at 24 h, suggesting that this lower concentration is insufficient for complete bacterial control.

Similarly, the inhibitory effect of compound **5u** on *P. aeruginosa* was evaluated and was found to be significantly concentration- and time-dependent (Figure 3b). At concentrations equal to or exceeding 1 MIC, the OD values for the 1 MIC and 2 MIC groups decreased to 0.064 and 0.057, respectively, from a control value of 0.301 (approximately 79% inhibition), and remained low at 24 h (1 MIC: 0.117; 2 MIC: 0.062). In contrast, the 0.5 MIC group showed limited efficacy with rebound tendencies; its OD value increased to 0.626 at 12 h, approaching the control value of 0.741, and remained elevated at 0.638 by 24 h, which may indicate bacterial adaptation. High concentrations (>1 MIC) demonstrated rapid and stable inhibition: the 1 MIC group achieved an OD value of 0.064 within 4 h, with only a modest increase to 0.117 by 24 h, while the 2 MIC group’s OD value at 24 h (0.062) even fell below the initial baseline, suggesting complete bacterial eradication or metabolic suppression. In brief, compound **5u** at concentrations of ≥1 MIC provided potent and durable inhibition against both *Salmonella* and *P. aeruginosa*, whereas a 0.5 MIC concentration only temporarily delayed growth without fully controlling bacterial proliferation.

### 2.3. Molecular Docking Comparison of Compound ***5u*** and Chalcone

**Figure 4:**
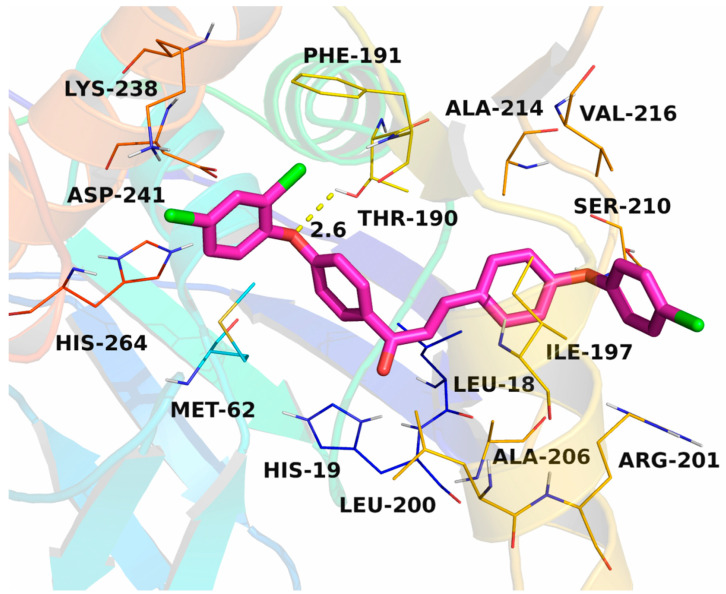
Compound **5u** was docked to the binding pocket of the LpxC (detailed view).

LpxC is a key and essential enzyme in the biosynthesis pathway of lipid A in Gram-negative bacteria [37]. In order to further verify the antibacterial activity of compound **5u**, the theoretical binding mode between **5u** and *P. aeruginosa* LpxC was investigated (Figure 4) [38,39,40]. The evaluated binding energy between **5u** and LpxC was −8.2 kcal mol^−1^. Compound **5u** adopted a W-shaped conformation in the pocket of the LpxC. Compound **5u** stretched into the hydrophobic pocket that consisted of the residues Leu-18, Met-62, Phe-191, Ile-197, Leu-200, Ala-206, Ala-214, and Val-216, forming strong hydrophobic bindings. Detailed analysis showed that the 2,4-dichlorophenyl group of **5u** formed a Cl-π interaction with the residue Phe-191, while the 4-chlorophenyl group of **5u** formed a cation-π interaction with the residue Arg-201. Importantly, one hydrogen bond interaction was observed between the residue Thr-190 and **5u** (bond length: 2.6 Å), which was the main interactions between **5u** and LpxC. All these interactions helped **5u** to anchor in the binding site of LpxC.

**Figure 5:**
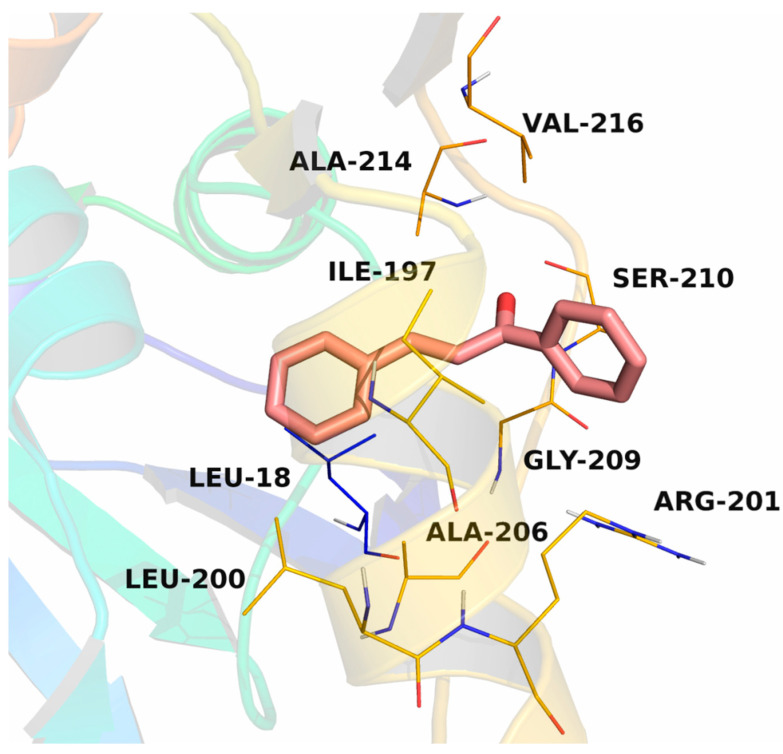
Chalcone was docked to the binding pocket of the LpxC (total view).

To further clarify the activity order between **5u** and chalcone, chalcone was docked to the binding pocket of the LpxC, and the theoretical binding mode between chalcone and LpxC was investigated (Figure 5). The evaluated binding energy between chalcone and LpxC was −7.2 kcal mol^−1^. Chalcone adopted a compact conformation in the pocket of the LpxC. Chalcone stretched into the hydrophobic pocket that consisted of the residues Leu-18, Ile-197, Leu-200, Ala-206, Ala-214, and Val-216, forming stable hydrophobic bindings. Detailed analysis showed that one of the phenyl groups of chalcone formed cation-π interaction with the residue Arg-201, which was the main interactions between chalcone and LpxC. All these interactions helped chalcone to anchor in the binding site of LpxC.

**Figure 6:**
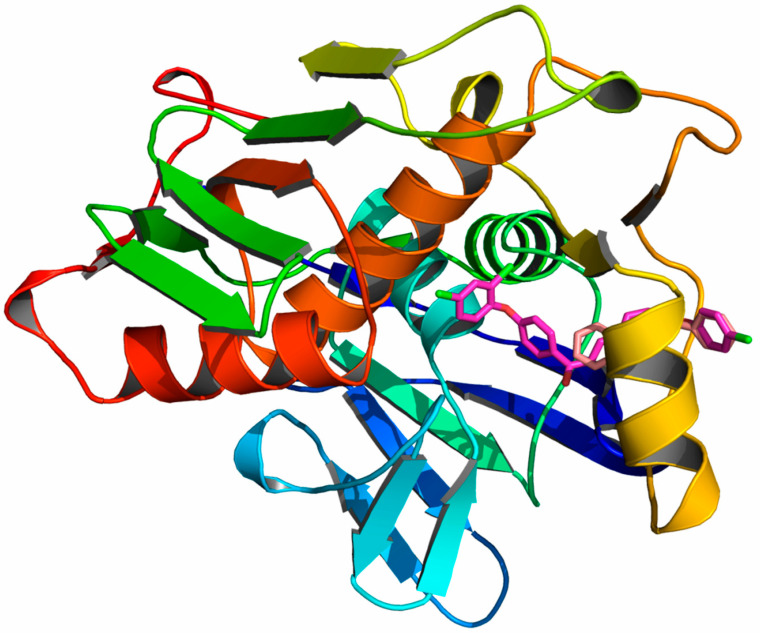
Chalcone and **5u** were docked to the binding pocket of the LpxC (overlapped).

Chalcone overlapped the half conformation of **5u** in the pocket of the LpxC (Figure 6). Regardless of hydrophobic interactions, π interactions, and hydrogen bond interactions, chalcone was weaker than compound **5u**, which made **5u** more active than chalcone against LpxC. This result is consistent with the antibacterial activity observed in the experiment.

### 2.4. Molecular Modeling of Compound ***5u***

**Figure 7:**
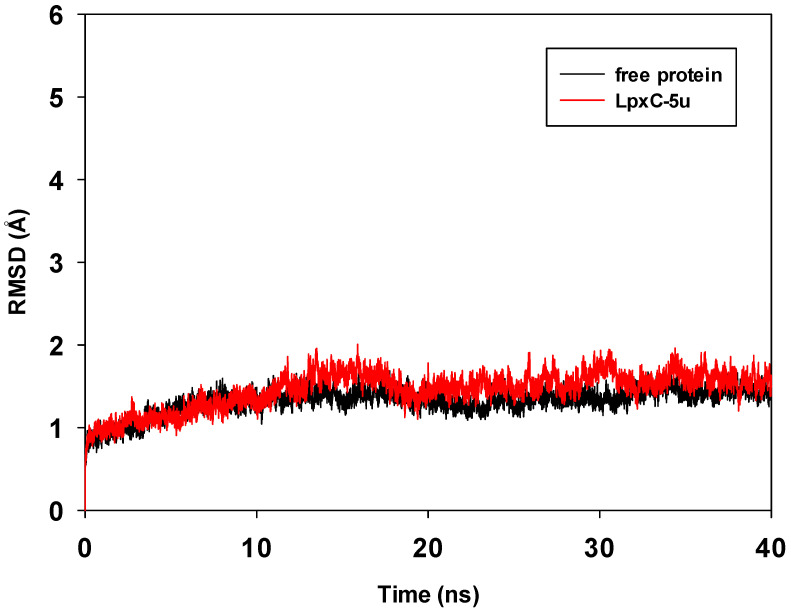
The root mean square deviations (RMSDs) of all the atoms of LpxC-**5u** complex with respect to its initial structure as function of time.

To further explore the potential binding mode between **5u** and LpxC, molecular dynamics simulations were performed using the AutoDock vina 1.1.2 and Amber14 software package [41,42,43,44,45,46,47]. The preferential binding mechanism of LpxC with **5u** was determined by 40 ns molecular dynamics simulations based on the docking results. To explore the dynamic stability of the models and to ensure the rationality of the sampling strategy, the root mean square deviation (RMSD) values of the protein backbone based on the starting structure along the simulation time were calculated and plotted in Figure 7 and the protein structures of the two systems were stabilized during the simulation.

**Figure 8:**
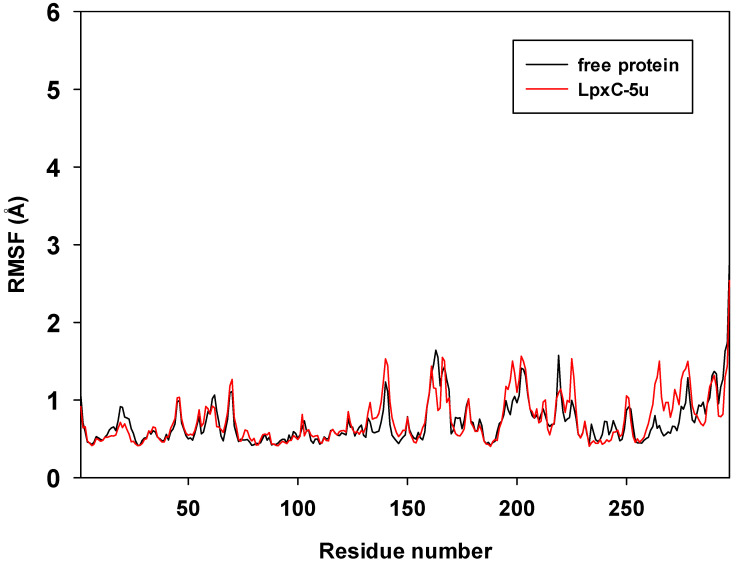
RMSF of residues of the whole protein in LpxC-**5u** complex and free LpxC during the 40 ns simulation.

The root mean square fluctuations (RMSF) of the residues of the whole protein in the LpxC-**5u** complex and in the free LpxC were calculated to reveal the flexibility of these residues. The RMSF of these residues are shown in Figure 8, clearly depicting different flexibilities in the binding site of LpxC in the presence and absence of **5u**. All of the residues in the LpxC binding site that bound with **5u** showed a small degree of flexibility with an RMSF of less than 2 Å when compared with free LpxC, indicating that these residues seemed to be more rigid as a result of binding to **5u**.

**Figure 9:**
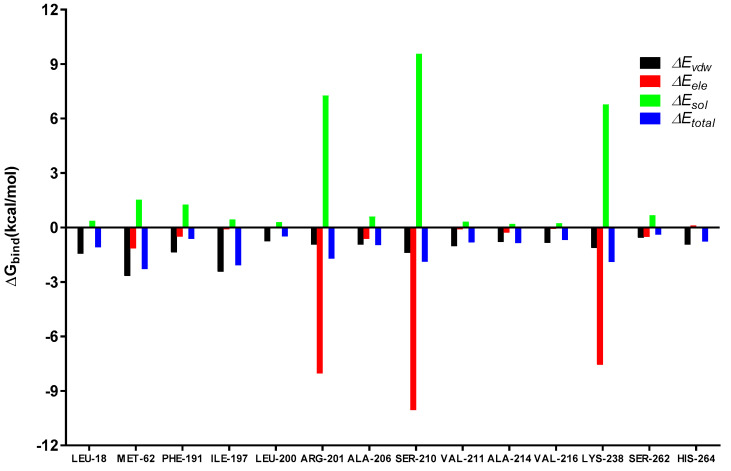
Decomposition of the binding energy on a per-residue basis in the LpxC-**5u** complex.

**Figure 10:**
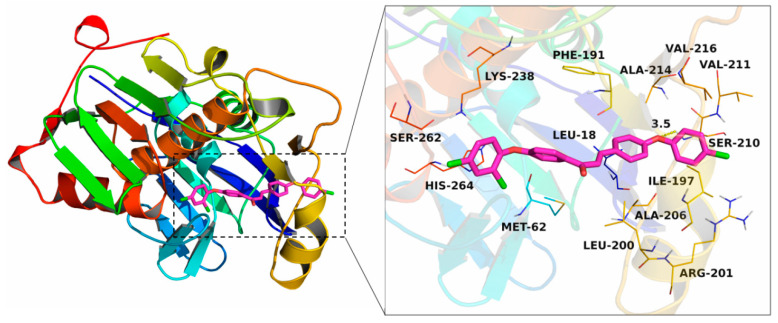
The predicted binding mode of **5u** in the LpxC binding pocket obtained from MD simulation.

To gain more information about the residues surrounding the binding site and their contribution to the whole system, the electrostatic, Van der Waals, solvation, and total contribution values of the residues to the binding free energy were calculated with the MMGBSA method. The summations of the per residue interaction free energies were separated into Van der Waals (∆Evdw), solvation (∆Esol), electrostatic (∆Eele), and total contribution (∆Etotal). In the LpxC-**5u** complex, the residue Ser-210 had a strong electrostatic (∆Eele) contribution, with an ∆Eele of <−10.0 kcal/mol (Figure 9). Detailed analysis showed that the residue Ser-210 formed a hydrogen bond interaction with **5u**, with a bond length of 3.5 Å (Figure 10). Moreover, the residues Arg-201 and Lys-238 had moderate electrostatic (∆Eele) contributions, with an ∆Eele of <−7.5 kcal/mol (Figure 9). Detailed analysis showed that the residues Arg-201 and Lys-238 formed cation-π interactions with phenyl groups of **5u** (Figure 10). In addition, residues Met-62 and Ile-197, with ∆Evdw values of <−2.0 kcal/mol, had strong Van der Waals interaction with the ligand because of the close proximity between the residue and the **5u**. Overall, the majority of the decomposed energy interaction originated from Van der Waals interactions, mainly through hydrophobic interactions, such as Leu-18, Phe-191, Leu-200, Ala-206, Val-211, Ala-214, and Val-216. In addition, the total binding free energy for the LpxC-**5u** complex calculated according to the MMGBSA approach, and an estimated ∆Gbind value of −38.0 kcal/mol was obtained for **5u**, suggesting that **5u** can bind to the binding site of the LpxC.

## 3. Materials and Methods

### 3.1. General Chemical Procedures

All starting materials and reagents were commercially available and were used without further purification. Merck silica gel GF_254_ plates (Darmstadt, Germany) were used for analytical TLC. ^1^H and ^13^C NMR spectra were obtained on a Bruker, AVAVNCE NEO 400 (Billerica, MA, USA, 400 MHz for ^1^H and 100 MHz for ^13^C) in DMSO-*d*_6_. Chemical shifts are expressed in δ (ppm) and coupling constants (*J*) in Hz. The high-resolution mass (HRMS) spectra were recorded on a Bruker maxis impact Q-TOF instrument (Bruker, Billerica, MA, USA) coupled with a Dionex Ultimate 3000 spectrometer (Dionex, Sunnyvale, CA, USA) and the melting points of the target compounds were determined on an X-4B melting point apparatus (Shanghai Precision Scientific Instrument Ltd., Shanghai, China). The FR-IR spectrum data were obtained on a Thermo Scientific Nicolet Summit X (Thermo Fisher Scientific, Waltham, MA, USA). The calculation of Lipinski’s rule of compounds’ five parameters was carried out using the XLOGP3^+^ software v1.0.0 [48].

### 3.2. Bacterial Strains

Bacterial strains of *S. aureus*, *E. coli*, *Salmonella*, and *P. aeruginosa* were provided by the Key Laboratory of Industrial Fermentation Microorganism of Chongqing University of Science and Technology.

### 3.3. General Procedure of for the Synthesis of Compounds ***5a***-***5u***

#### 3.3.1. Synthesis of Intermediate Compound **3**

Compound **1** (15 mmol) was dissolved in DMSO (10 mL), and then Cs_2_CO_3_ (10 mmol) was added, and finally compound **2** (10 mmol) was added into the solution. The reaction mixture was stirred at 150 °C, and monitored by TLC (petroleum ether/ethyl acetate 20:1). After the reaction was completed at 8 h, the solvent was removed under reduced pressure and the residue was purified by column chromatography with petroleum ether/ethyl acetate (40:1) as eluent to give intermediate compound **3** with 76% yield.

#### 3.3.2. Synthesis of Target Compounds **5a**-**5u**

Compound **4**, (20 mmol), NaOH solution (10%, 4 mL), and intermediate compound **3** (20 mmol) were dissolved in anhydrous ethanol (10 mL). The mixture was stirred for 5–10 h at room temperature, and monitored by TLC (petroleum ether/ethyl acetate 30:1). Ice water (100 mL) was added and the mixture was neutralized with HCl (5%). The solid was precipitated out, filtered off, and washed with water. The pure target compounds **5a**-**5u** were obtained by recrystallization in anhydrous ethanol. The chemical structures of the target compounds were accurately confirmed using ^1^H NMR, ^13^C NMR, HRMS, and FR-IR. The yields and spectral data of target compounds **5a**-**5u** are given below.

*(E)-1-(4-(2,4-Dichlorophenoxy)phenyl)-3-(2-fluorophenyl)prop-2-en-1-one* **5a**, yellow powder, m.p. 110–112 °C, Yield: 83%. IR(KBr): ʋ_max_ 3069, 1664, 1605, 1504, 1280, 1258, 975, 868, 822, 750, 697, 708 cm^−1^; ^1^H NMR (400 MHz, DMSO-*d*_6_) δ 8.21 (dt, *J* = 9.2, 2.9 Hz, 2H), 8.16–8.08 (m, 1H), 8.04–7.94 (m, 1H), 7.86 (dt, *J* = 6.1, 2.7 Hz, 1H), 7.84–7.78 (m, 1H), 7.54 (dd, *J* = 6.3, 2.7 Hz, 1H), 7.52–7.44 (m, 1H), 7.38–7.35 (m, 1H), 7.35–7.29 (m, 1H), 7.11 (d, *J* = 2.9 Hz, 1H), 7.09 (t, *J* = 2.7 Hz, 2H); ^13^C NMR (100 MHz, DMSO-*d*_6_) δ 187.87 (s, 1C), 161.39 (d, 1C), 161.00 (s, 1C), 149.68 (s, 1C), 135.38 (d, 1C), 133.15 (d, 1C), 133.00 (s, 1C), 131.79 (s, 2C), 130.92 (s, 1C), 130.48 (s, 1C), 129.67 (d, 1C), 127.14 (s, 1C), 125.46 (d, 1C), 124.46 (d, 1C), 122.77 (d, 1C), 122.23 (s, 1C), 118.09 (s, 1C), 117.03 (s, 2C), 116.57 (d, 1C). ESI-HRMS: *m*/*z* [M + H]^+^ calcd. for C_21_H_14_Cl_2_FO_2_ 387.0355, found 387.0358.

*(E)-3-(2-Chlorophenyl)-1-(4-(2,4-dichlorophenoxy)phenyl)prop-2-en-1-one* **5b**, yellow powder, m.p. 117–119 °C, Yield: 86%. IR (KBr): ʋ_max_ 3063, 1681, 1594, 1502, 1308, 1254, 979, 867, 829, 749, 685, 705 cm^−1^; ^1^H NMR (400 MHz, DMSO-*d*_6_) δ 8.25 (d, *J* = 2.0 Hz, 1H), 8.23 (q, *J* = 2.8 Hz, 2H), 8.10–7.96 (m, 2H), 7.86 (d, *J* = 2.5 Hz, 1H), 7.60–7.56 (m, 1H), 7.54 (dd, *J* = 8.7, 2.5 Hz, 1H), 7.50–7.47 (m, 1H), 7.48–7.44 (m, 1H), 7.36 (d, *J* = 8.7 Hz, 1H), 7.15–7.04 (m, 2H); ^13^C NMR (100 MHz, DMSO-*d*_6_) δ 187.80 (s, 1C), 161.03 (s, 1C), 149.68 (s, 1C), 138.76 (s, 1C), 134.83 (s, 1C), 132.96 (s, 1C), 132.76 (s, 1C), 132.45 (s, 1C), 131.87 (s, 2C), 130.91 (s, 1C), 130.51 (s, 1C), 130.48 (s, 1C), 129.70 (s, 1C), 129.07 (s, 1C), 128.15 (s, 1C), 127.14 (s, 1C), 125.06 (s, 1C), 124.45 (s, 1C), 117.00 (s, 2C). ESI-HRMS: *m*/*z* [M + H]^+^ calcd. for C_21_H_14_Cl_3_O_2_ 403.0059, found 403.0063.

*(E)-3-(2-Bromophenyl)-1-(4-(2,4-dichlorophenoxy)phenyl)prop-2-en-1-one* **5c**, yellow powder, m.p. 119–121 °C, Yield: 87%. IR (KBr): ʋ_max_ 3060, 1659, 1596, 1503, 1310, 1240, 973, 867, 828, 749, 702, 660 cm^−1^; ^1^H NMR (400 MHz, DMSO-*d*_6_) δ 8.28–8.22 (m, 2H), 8.20 (dd, *J* = 7.9, 1.8 Hz, 1H), 7.99 (d, *J* = 4.5 Hz, 2H), 7.87 (d, *J* = 2.6 Hz, 1H), 7.75 (dd, *J* = 8.0, 1.3 Hz, 1H), 7.60–7.52 (m, 1H), 7.49 (dd, *J* = 7.6, 1.3 Hz, 1H), 7.41 (dd, *J* = 7.7, 1.7 Hz, 1H), 7.36 (d, *J* = 8.8 Hz, 1H), 7.11 (d, *J* = 2.1 Hz, 1H), 7.09 (d, *J* = 2.0 Hz, 1H); ^13^C NMR (100 MHz, DMSO-*d*_6_) δ 187.80 (s, 1C), 161.04 (s, 1C), 149.67 (s, 1C), 141.51 (s, 1C), 134.43 (s, 1C), 133.78 (s, 1C), 132.96 (s, 1C), 132.65 (s, 1C), 131.89 (s, 2C), 130.92 (s, 1C), 130.48 (s, 1C), 129.72 (s, 1C), 129.25 (s, 1C), 128.71 (s, 1C), 127.14 (s, 1C), 125.86 (s, 1C), 125.23 (s, 1C), 124.48 (s, 1C), 117.01 (s, 2C). ESI-HRMS: *m*/*z* [M + H]^+^ calcd. for C_21_H_14_BrCl_2_O_2_ 446.9554, found 446.9556.

*(E)-1-(4-(2,4-Dichlorophenoxy)phenyl)-3-(o-tolyl)prop-2-en-1-one* **5d**, light yellow powder, m.p. 105–107 °C, Yield: 81%. IR (KBr): ʋ_max_ 3060, 2971, 1656, 1592, 1482, 1379, 1318, 1253, 978, 867, 827, 755, 698, 669 cm^−1^; ^1^H NMR (400 MHz, DMSO-*d*_6_) δ 8.22 (dq, *J* = 9.2, 2.4 Hz, 2H), 8.05–8.00 (m, 1H), 7.99 (d, *J* = 2.0 Hz, 1H), 7.89–7.84 (m, 1H), 7.83 (dq, *J* = 4.6, 2.1 Hz, 1H), 7.55–7.49 (m, 1H), 7.35 (d, *J* = 2.1 Hz, 1H), 7.34 (d, *J* = 1.5 Hz, 1H), 7.30 (d, *J* = 6.6 Hz, 1H), 7.28 (d, *J* = 1.7 Hz, 1H), 7.14–7.04 (m, 2H), 2.45 (s, 3H); 13C NMR (100 MHz, DMSO-*d*_6_) δ 193.76 (s, 1C), 188.00 (s, 1C), 160.81 (s, 1C), 149.74 (s, 1C), 141.27 (s, 1C), 138.45 (s, 1C), 133.77 (s, 1C), 131.70 (s, 2C), 131.25 (s, 1C), 130.87 (s, 1C), 130.80 (s, 1C), 129.64 (s, 1C), 127.32 (s, 1C), 127.12 (s, 1C), 126.83 (s, 1C), 124.35 (s, 1C), 123.15 (s, 1C), 122.13 (s, 1C), 116.96 (s, 2C), 19.81 (s, 1C). ESI-HRMS: *m*/*z* [M + H]^+^ calcd. for C_22_H_17_Cl_2_O_2_ 383.0606, found 383.0608.

*(E)-1-(4-(2,4-Dichlorophenoxy)phenyl)-3-(2-methoxyphenyl)prop-2-en-1-one* **5e**, orange powder, m.p. 107–109 °C, Yield: 84%. IR (KBr): ʋ_max_ 3071, 2962, 1653, 1599, 1435, 1414, 1335, 1315, 1241, 970, 870, 825, 745, 702, 670 cm^−1^; ^1^H NMR (400 MHz, DMSO-*d*_6_) δ 8.28–8.14 (m, 2H), 8.07 (d, *J* = 15.7 Hz, 1H), 8.01–7.94 (m, 1H), 7.90 (d, *J* = 15.7 Hz, 1H), 7.85 (d, *J* = 2.5 Hz, 1H), 7.53 (dt, *J* = 8.9, 2.7 Hz, 1H), 7.46 (ddd, *J* = 8.7, 7.3, 1.6 Hz, 1H), 7.35 (d, *J* = 8.7 Hz, 1H), 7.16–7.12 (m, 1H), 7.12–7.06 (m, 2H), 7.04 (d, *J* = 7.4 Hz, 1H), 3.91 (s, 3H); ^13^C NMR (100 MHz, DMSO-*d*_6_) δ 188.14 (s, 1C), 160.73 (s, 1C), 158.72 (s, 1C), 149.76 (s, 1C), 138.80 (s, 1C), 132.76 (s, 1C), 131.59 (s, 2C), 130.88 (s, 1C), 130.40 (s, 1C), 129.66 (s, 1C), 129.02 (s, 1C), 127.12 (s, 1C), 124.38 (s, 1C), 123.40 (s, 1C), 122.10 (s, 1C), 121.15 (s, 1C), 118.04 (s, 1C), 116.97 (s, 2C), 112.23 (s, 1C), 56.16 (s, 1C). ESI-HRMS: *m*/*z* [M + H]^+^ calcd. for C_22_H_17_Cl_2_O_3_ 399.0555, found 399.0557.

*(E)-1-(4-(2,4-Dichlorophenoxy)phenyl)-3-(2-(trifluoromethyl)phenyl)prop-2-en-1-one* **5f**, light yellow powder, m.p. 120–122 °C, Yield: 82%. IR (KBr): ʋ_max_ 2972, 1663, 1601, 1576, 1483, 1416, 1384, 1330, 1311, 1280, 974, 872, 829, 751, 703, 651 cm^−1^; ^1^H NMR (400 MHz, DMSO-*d*_6_) δ 8.34 (d, *J* = 7.2 Hz, 1H), 8.31–8.16 (m, 2H), 8.13–8.03 (m, 1H), 7.98 (d, *J* = 14.5 Hz, 1H), 7.86 (dd, *J* = 9.9, 4.6 Hz, 1H), 7.81 (t, *J* = 6.8 Hz, 1H), 7.68 (t, *J* = 7.1 Hz, 1H), 7.54 (ddt, *J* = 9.1, 6.6, 3.3 Hz, 1H), 7.38 (dd, *J* = 8.9, 4.9 Hz, 1H), 7.18–7.07 (m, 2H), 7.08–6.99 (m, 1H); ^13^C NMR (100 MHz, DMSO-*d*_6_) δ 187.67 (s, 1C), 161.13 (s, 1C), 149.64 (s, 1C), 138.02 (s, 1C), 133.43 (s, 1C), 132.77 (s, 1C), 131.95 (s, 2C), 130.96 (s, 1C), 130.91 (s, 1C), 130.69 (s, 1C), 130.51 (s, 1C), 129.70 (s, 1C), 129.26 (s, 1C), 127.15 (s, 1C), 126.64 (dd, *J* = 6 Hz, 1C), 126.51 (s, 1C), 124.47 (s, 1C), 122.22 (s, 1C), 118.05 (s, 1C), 117.00 (s, 2C). ESI-HRMS: *m*/*z* [M + H]^+^ calcd. for C_22_H_14_Cl_2_F_3_O_2_ 437.0323, found 437.0325.

*(E)-1-(4-(2,4-Dichlorophenoxy)phenyl)-3-(3-fluorophenyl)prop-2-en-1-one* **5g**, light yellow powder, m.p. 114–116 °C, Yield: 80%. IR (KBr): ʋ_max_ 2972, 1664, 1599, 1502, 1304, 1234, 979, 865, 805, 752, 691, 691 cm^−1^; ^1^H NMR (400 MHz, DMSO-*d*_6_) δ 8.32–8.14 (m, 2H), 8.02 (dd, *J* = 15.7, 3.4 Hz, 1H), 7.89–7.85 (m, 1H), 7.85 (s, 1H), 7.76 (d, *J* = 3.4 Hz, 1H), 7.73–7.67 (m, 1H), 7.52 (s, 1H), 7.51 (s, 1H), 7.35 (dd, *J* = 8.7, 3.5 Hz, 1H), 7.33–7.23 (m, 1H), 7.10 (dd, *J* = 8.6, 3.5 Hz, 2H); ^13^C NMR (100 MHz, DMSO-*d*_6_) δ 187.95 (s, 1C), 162.97 (d, *J* = 242 Hz, 1C), 160.94 (s, 1C), 149.72 (s, 1C), 142.78 (d, *J* = 3 Hz, 1C), 137.75 (d, *J* = 7 Hz, 1C), 133.11 (s, 1C), 131.83 (s, 2C), 131.32(d, *J* = 9 Hz, 1C), 130.90 (s, 1C), 130.44 (s, 1C), 129.69 (s, 1C), 127.11 (s, 1C), 126.07 (s, 1C), 124.41 (s, 1C), 123.79 (s, 1C), 117.72 (d, *J* = 22 Hz, 1C), 116.99 (s, 2C), 115.14 (d, *J* = 22 Hz, 1C). ESI-HRMS: *m*/*z* [M + H]^+^ calcd. for C_21_H_14_Cl_2_FO_2_ 387.0355, found 387.0357.

*(E)-3-(3-Chlorophenyl)-1-(4-(2,4-dichlorophenoxy)phenyl)prop-2-en-1-one* **5h**, light yellow powder, m.p. 120–122 °C, Yield: 80%. IR (KBr): ʋ_max_ 3097, 1663, 1594, 1503, 1311, 1262, 976, 869, 814, 750, 685, 685 cm^−1^; ^1^H NMR (400 MHz, DMSO-*d*_6_) δ 8.29–8.20 (m, 2H), 8.09 (d, *J* = 2.0 Hz, 1H), 8.08–8.00 (m, 1H), 7.87 (dd, *J* = 2.5, 0.9 Hz, 1H), 7.83 (dt, *J* = 6.6, 1.9 Hz, 1H), 7.72 (d, *J* = 15.6 Hz, 1H), 7.57–7.52 (m, 1H), 7.51 (q, *J* = 1.5 Hz, 1H), 7.48 (d, *J* = 7.9 Hz, 1H), 7.36 (d, *J* = 8.7 Hz, 1H), 7.14–7.03 (m, 2H); ^13^C NMR (100 MHz, DMSO-*d*_6_) δ 187.93 (s, 1C), 160.96 (s, 1C), 149.72 (s, 1C), 142.54 (s, 1C), 137.44 (s, 1C), 134.27 (s, 1C), 133.10 (s, 1C), 131.87 (s, 2C), 131.17 (s, 1C), 130.92 (s, 1C), 130.61 (s, 1C), 130.45 (s, 1C), 129.71 (s, 1C), 128.43 (s, 1C), 128.40 (s, 1C), 127.12 (s, 1C), 124.44 (s, 1C), 123.89 (s, 1C), 116.99 (s, 2C). ESI-HRMS: *m*/*z* [M + H]^+^ calcd. for C_21_H_14_Cl_3_O_2_ 403.0059, found 403.0063.

*(E)-3-(3-Bromophenyl)-1-(4-(2,4-dichlorophenoxy)phenyl)prop-2-en-1-one* **5i**, yellow powder, m.p. 124–126 °C, Yield: 84%. IR (KBr): ʋ_max_ 3403, 1652, 1601, 1557, 1307, 1241, 943, 867, 817, 752, 690, 662 cm^−1^; ^1^H NMR (400 MHz, DMSO-*d*_6_) δ 8.26 (t, *J* = 2.8 Hz, 1H), 8.23 (dd, *J* = 6.1, 3.1 Hz, 2H), 8.04 (d, *J* = 15.6 Hz, 1H), 7.87 (d, *J* = 3.3 Hz, 1H), 7.86–7.80 (m, 1H), 7.71 (dt, *J* = 15.6, 2.9 Hz, 1H), 7.64 (dt, *J* = 8.2, 2.6 Hz, 1H), 7.53 (dt, *J* = 8.8, 3.1 Hz, 1H), 7.45–7.30 (m, 2H), 7.10 (q, *J* = 3.1 Hz, 1H), 7.09–7.02 (m, 1H); ^13^C NMR (100 MHz, DMSO-*d*_6_) δ 187.90 (s, 1C), 160.95 (s, 1C), 149.72 (s, 1C), 142.49 (s, 1C), 137.69 (s, 1C), 133.49 (s, 1C), 133.10 (s, 1C), 131.87 (s, 2C), 131.42 (s, 1C), 131.28 (s, 1C), 130.91 (s, 1C), 130.44 (s, 1C), 129.70 (s, 1C), 128.77 (s, 1C), 127.12 (s, 1C), 124.43 (s, 1C), 123.85 (s, 1C), 122.88 (s, 1C), 116.98 (s, 2C). ESI-HRMS: *m*/*z* [M + H]^+^ calcd. for C_21_H_14_BrCl_2_O_2_ 446.9554, found 446.9556.

*(E)-1-(4-(2,4-Dichlorophenoxy)phenyl)-3-(m-tolyl)prop-2-en-1-one* **5j**, yellow powder, m.p. 108–110 °C, Yield: 80%. IR (KBr): ʋ_max_ 3680, 2920, 1660, 1595, 1470, 1379, 1314, 1255, 978, 861, 817, 751, 692, 666 cm^−1^; ^1^H NMR (400 MHz, DMSO-*d*_6_) δ 8.26–8.18 (m, 2H), 7.98–7.88 (m, 1H), 7.75–7.71 (m, 1H), 7.69–7.64 (m, 1H), 7.53 (s, 1H), 7.51 (s, 1H), 7.36 (ddd, *J* = 9.4, 6.7, 2.0 Hz, 1H), 7.28 (d, *J* = 7.5 Hz, 1H), 7.20 (q, *J* = 2.0 Hz, 1H), 7.19 (t, *J* = 2.0 Hz, 1H), 7.16–7.10 (m, 2H), 2.37 (s, 3H); ^13^C NMR (100 MHz, DMSO-*d*_6_) δ 188.06 (s, 1C), 161.27 (s, 1C), 154.53 (s, 1C), 144.40 (s, 1C), 138.64 (s, 1C), 135.08 (s, 1C), 133.22 (s, 1C), 131.81 (s, 1C),131.70 (s, 1C), 131.67 (s, 1C), 130.91 (s, 1C), 130.69 (s, 2C), 129.62 (s, 1C), 129.29 (s, 1C), 129.03 (s, 1C), 126.80 (s, 1C), 122.16 (s, 1C), 122.12 (s, 1C), 118.07 (s, 2C), 21.35 (s, 1C). ESI-HRMS: *m*/*z* [M + H]^+^ calcd. for C_22_H_17_Cl_2_O_2_ 383.0606, found 383.0608.

*(E)-1-(4-(2,4-Dichlorophenoxy)phenyl)-3-(3-methoxyphenyl)prop-2-en-1-one* **5k**, light yellow powder, m.p. 110–112 °C, Yield: 85%. IR (KBr): ʋ_max_ 3065, 2937, 1660, 1598, 1470, 1432, 1415, 1291, 1235, 979, 870, 828, 733, 693, 670 cm^−1^; ^1^H NMR (400 MHz, DMSO-*d*_6_) δ 8.28–8.17 (m, 2H), 7.95 (ddt, *J* = 15.5, 6.3, 3.1 Hz, 1H), 7.86 (p, *J* = 3.5 Hz, 1H), 7.72 (dd, *J* = 15.5, 2.6 Hz, 1H), 7.50–7.47 (m, 1H), 7.46–7.41 (m, 1H), 7.40–7.36 (m, 1H), 7.36–7.31 (m, 1H), 7.22–7.13 (m, 1H), 7.08 (ddt, *J* = 12.9, 8.9, 3.1 Hz, 2H), 7.03 (dd, *J* = 5.6, 2.8 Hz, 1H), 3.84 (s, 3H); ^13^C NMR (100 MHz, DMSO-*d*_6_) δ 188.10 (s, 1C), 160.84 (s, 1C), 160.11 (s, 1C), 149.75 (s, 1C), 144.28 (s, 1C), 136.56 (s, 1C), 133.27 (s, 1C), 131.76 (s, 1C), 130.90 (s, 1C), 130.69 (s, 1C), 130.42 (s, 2C), 129.70 (s, 1C), 127.11 (s, 1C), 124.41 (s, 1C), 122.63 (s, 1C), 122.15 (s, 1C), 117.14 (s, 1C), 116.98 (s, 2C), 113.87 (s, 1C), 55.77 (s, 1C). ESI-HRMS: *m*/*z* [M + H]^+^ calcd. for C_22_H_17_Cl_2_O_3_ 399.0555, found 399.0557.

*(E)-1-(4-(2,4-Dichlorophenoxy)phenyl)-3-(3-(trifluoromethyl)phenyl)prop-2-en-1-one* **5l**, light yellow powder, m.p. 124–126 °C, Yield: 83%. IR (KBr): ʋ_max_ 3068, 1665, 1601, 1578, 1483, 1417, 1327, 1237, 1214, 984, 869, 830, 751, 702, 658 cm^−1^; ^1^H NMR (400 MHz, DMSO-*d*_6_) δ 8.47–8.23 (m, 1H), 8.14 (d, *J* = 29.0 Hz, 1H), 8.01–7.85 (m, 1H), 7.85–7.74 (m, 1H), 7.75–7.61 (m, 1H), 7.60–7.44 (m, 2H), 7.43–7.25 (m, 2H), 7.18 (d, *J* = 21.3 Hz, 1H), 7.15–7.06 (m, 1H), 7.06–6.96 (m, 1H), 6.82 (s, 1H); ^13^C NMR (100 MHz, DMSO-*d*_6_) δ 187.98 (s, 1C), 160.98 (s, 1C), 149.72 (s, 1C), 142.41 (s, 1C), 136.35 (s, 1C), 133.34 (s, 1C), 131.91 (s, 1C), 130.90 (s, 1C), 130.68 (s, 1C), 130.45 (s, 1C), 130.40 (s, 2C), 129.69 (s, 1C), 127.12 (s, 1C), 125.58 (s, 1C), 124.90 (s, 1C), 124.41 (s, 1C), 123.24 (d, J = 215 Hz, 1C), 118.25 (s, 1C), 118.05 (s, 1C), 116.98 (s, 2C). ESI-HRMS: *m*/*z* [M + H]^+^ calcd. for C_22_H_14_Cl_2_F_3_O_2_ 437.0323, found 437.0326.

*(E)-1-(4-(2,4-Dichlorophenoxy)phenyl)-3-(4-fluorophenyl)prop-2-en-1-one* **5m**, light yellow powder, m.p. 120–122 °C, Yield: 85%. IR (KBr): ʋ_max_ 3066, 1655, 1597, 1504, 1301, 1234, 991, 870, 813, 757, 699, 659 cm^−1^; ^1^H NMR (400 MHz, DMSO-*d*_6_) δ 8.29–8.14 (m, 2H), 7.97 (qd, *J* = 7.4, 3.3 Hz, 2H), 7.92–7.81 (m, 1H), 7.75 (dd, *J* = 15.6, 3.9 Hz, 1H), 7.52 (dp, *J* = 10.0, 3.3 Hz, 1H), 7.36–7.28 (m, 2H), 7.18 (dt, *J* = 10.3, 3.7 Hz, 1H), 7.09 (ddq, *J* = 10.2, 6.5, 3.4 Hz, 2H), 7.03 (dt, *J* = 7.7, 4.3 Hz, 1H); ^13^C NMR (100 MHz, DMSO-*d*_6_) δ 187.97 (s, 1C), 163.92 (d, 1C), 160.83 (s, 1C), 149.73 (s, 1C), 143.04 (s, 1C), 131.85 (d, 1C), 131.76 (s, 1C), 131.72 (s, 2C), 131.68 (s, 2C), 130.89 (s, 1C), 130.68 (s, 1C), 129.69 (s, 1C), 124.41 (s, 1C), 122.16 (s, 1C), 118.04 (s, 1C), 116.97 (s, 2C), 116.52 (s, 1C), 116.30 (s, 1C). ESI-HRMS: *m*/*z* [M + H]^+^ calcd. for C_21_H_14_Cl_2_FO_2_ 387.0355, found 387.0353.

*(E)-3-(4-Chlorophenyl)-1-(4-(2,4-dichlorophenoxy)phenyl)prop-2-en-1-one* **5n**, light yellow powder, m.p. 125–127 °C, Yield: 80%. IR (KBr): ʋ_max_ 3066, 1678, 1599, 1502, 1317, 1255, 978, 867, 867, 752, 705, 658 cm^−1^; ^1^H NMR (400 MHz, DMSO-*d*_6_) δ 8.22 (dd, *J* = 8.9, 3.4 Hz, 2H), 7.99 (s, 1H), 7.94 (d, *J* = 8.5 Hz, 2H), 7.86 (d, *J* = 2.5 Hz, 1H), 7.73 (d, *J* = 15.6 Hz, 1H), 7.55–7.52 (m, 2H), 7.35 (d, *J* = 8.8 Hz, 1H), 7.22–7.12 (m, 1H), 7.12–7.06 (m, 2H); ^13^C NMR (100 MHz, DMSO-*d*_6_) δ 187.97 (s, 1C), 160.91 (s, 1C), 149.72 (s, 1C), 142.80 (s, 1C), 135.55 (s, 1C), 134.14 (s, 1C), 133.16 (s, 1C), 131.78 (s, 2C), 131.06 (s, 2C), 130.91 (s, 1C), 129.71 (s, 1C), 129.44 (s, 2C), 127.13 (s, 1C), 124.45 (s, 1C), 123.09 (s, 1C), 118.06 (s, 1C), 116.98 (s, 2C). ESI-HRMS: *m*/*z* [M + H]^+^ calcd. for C_21_H_14_Cl_3_O_2_ 403.0059, found 403.0058.

*(E)-3-(4-Bromophenyl)-1-(4-(2,4-dichlorophenoxy)phenyl)prop-2-en-1-one* **5o**, light yellow powder, m.p. 130–132 °C, Yield: 86%. ^1^H NMR (400 MHz, DMSO-*d*_6_) δ 8.26–8.18 (m, 1H), 8.04–7.95 (m, 2H), 7.85 (tt, *J* = 5.4, 2.6 Hz, 2H), 7.76–7.68 (m, 1H), 7.67 (dd, *J* = 8.5, 2.8 Hz, 1H), 7.51 (ddt, *J* = 8.3, 5.4, 2.5 Hz, 1H), 7.33 (ddd, *J* = 11.7, 8.7, 2.8 Hz, 1H), 7.13 (qd, *J* = 25.2, 11.5, 7.2, 2.7 Hz, 2H), 7.07–6.95 (m, 2H). ESI-HRMS: *m*/*z* [M + H]^+^ calcd. for C_21_H_14_BrCl_2_O_2_ 446.9554, found 446.9555.

*(E)-1-(4-(2,4-Dichlorophenoxy)phenyl)-3-(p-tolyl)prop-2-en-1-one* **5p**, orange powder, m.p. 108–110 °C, Yield: 84%. IR (KBr): ʋ_max_ 3062, 2916, 1654, 1593, 1469, 1380, 1303, 1253, 982, 857, 838, 749, 696, 670 cm-1; ^1^H NMR (400 MHz, DMSO-*d*_6_) δ 8.20 (dt, *J* = 9.5, 3.0 Hz, 2H), 7.89–7.83 (m, 1H), 7.78 (d, *J* = 8.2 Hz, 2H), 7.72 (d, *J* = 15.7 Hz, 1H), 7.56–7.47 (m, 1H), 7.34 (dd, *J* = 8.8, 3.7 Hz, 1H), 7.27 (d, *J* = 8.2 Hz, 2H), 7.21–7.11 (m, 1H), 7.11–6.99 (m, 2H), 3.40 (s, 3H); ^13^C NMR (100 MHz, DMSO-*d*_6_) δ 188.02 (s, 1C), 160.74 (s, 1C), 149.78 (s, 1C), 144.35 (s, 1C), 141.15 (s, 1C), 133.39 (s, 1C), 132.44 (s, 1C), 131.65 (s, 2C), 130.89 (s, 1C), 130.67 (s, 1C), 130.02 (s, 2C), 129.68 (s, 1C), 129.39 (s, 1C), 127.10 (s, 1C), 124.38 (s, 1C), 122.14 (s, 1C), 121.27 (s, 1C), 116.98 (s, 2C), 21.57 (s, 1C). ESI-HRMS: *m*/*z* [M + H]^+^ calcd. for C_22_H_17_Cl_2_O_2_ 383.0606, found 383.0608.

*(E)-1-(4-(2,4-Dichlorophenoxy)phenyl)-3-(4-methoxyphenyl)prop-2-en-1-one* **5q**, yellow powder, m.p. 114–116 °C, Yield: 85%. IR (KBr): ʋ_max_ 3085, 2910, 1675, 1598, 1470, 1413, 1386, 1293, 1232, 959, 881, 832, 751, 697, 670 cm^−1^; ^1^H NMR (400 MHz, DMSO-*d*_6_) δ 8.25–8.14 (m, 2H), 7.85 (d, *J* = 2.0 Hz, 1H), 7.85–7.82 (m, 2H), 7.80 (d, *J* = 15.5 Hz, 1H), 7.72 (d, *J* = 15.5 Hz, 1H), 7.52 (dd, *J* = 8.7, 2.6 Hz, 1H), 7.34 (d, *J* = 8.7 Hz, 1H), 7.12–7.06 (m, 2H), 7.04–6.98 (m, 2H), 3.83 (s, 3H); ^13^C NMR (100 MHz, DMSO-*d*_6_) δ 187.92 (s, 1C), 161.83 (s, 1C), 160.64 (s, 1C), 149.82 (s, 1C), 144.28 (s, 1C), 133.58 (s, 1C), 131.56 (s, 2C), 131.27 (s, 2C), 130.90 (s, 1C), 130.35 (s, 1C), 129.68 (s, 1C), 127.79 (s, 1C), 127.08 (s, 1C), 124.35 (s, 1C), 119.81 (s, 1C), 116.98 (s, 2C), 116.78 (s, 1C), 114.88 (s, 2C). ESI-HRMS: *m*/*z* [M + H]^+^ calcd. for C_22_H_17_Cl_2_O_3_ 399.0555, found 399.0554.

*(E)-1-(4-(2,4-Dichlorophenoxy)phenyl)-3-(4-(trifluoromethyl)phenyl)prop-2-en-1-one* **5r**, light yellow powder, m.p. 132–134 °C, Yield: 81%. IR (KBr): ʋ_max_ 3070, 1677, 1601, 1578, 1483, 1416, 1323, 1234, 1161, 989, 870, 830, 750, 673, 622 cm^−1^; ^1^H NMR (400 MHz, DMSO-*d*_6_) δ 7.98 (ddd, *J* = 9.1, 4.9, 2.6 Hz, 2H), 7.84 (t, *J* = 2.5 Hz, 1H), 7.58 (d, *J* = 3.5 Hz, 1H), 7.54–7.48 (m, 2H), 7.31 (dd, *J* = 8.8, 5.7 Hz, 2H), 7.18–7.13 (m, 1H), 7.08 (d, *J* = 8.8 Hz, 1H), 7.05–7.01 (m, 2H), 7.00–6.97 (m, 1H). ESI-HRMS: *m*/*z* [M + H]^+^ calcd. for C_22_H_13_Cl_2_F_3_NaO_2_ 459.0142, found 459.0149.

*(E)-1-(4-(2,4-Dichlorophenoxy)phenyl)-3-(3-nitrophenyl)prop-2-en-1-one* **5s**, yellow powder, m.p. 125–127 °C, Yield: 87%. IR (KBr): ʋ_max_ 3071, 1652, 1599, 1570, 1484, 1418, 1348, 1221, 1169, 984, 874, 837, 745, 677, 640 cm^−1^; ^1^H NMR (400 MHz, DMSO-*d*_6_) δ 8.79 (s, 1H), 8.34 (d, *J* = 5.7 Hz, 1H), 8.28 (d, *J* = 3.0 Hz, 1H), 8.26 (q, *J* = 3.2 Hz, 2H), 8.17 (dt, *J* = 15.6, 3.0 Hz, 1H), 7.86 (ddd, *J* = 12.3, 6.9, 3.4 Hz, 1H), 7.75 (td, *J* = 8.0, 3.5 Hz, 1H), 7.53 (dq, *J* = 10.2, 3.3 Hz, 1H), 7.36 (dt, *J* = 8.8, 3.4 Hz, 1H), 7.22–7.14 (m, 1H), 7.11 (dt, *J* = 6.5, 4.7 Hz, 2H); ^13^C NMR (100 MHz, DMSO-*d*_6_) δ 187.93 (s, 1C), 161.03 (s, 1C), 149.69 (s, 1C), 148.88 (s, 1C), 141.71 (s, 1C), 137.07 (s, 1C), 135.58 (s, 1C), 131.93 (s, 2C),130.91 (s, 1C), 130.83 (s, 1C), 130.69 (s, 1C), 129.70 (s, 1C), 127.13 (s, 1C), 125.12 (s, 1C), 124.44 (s, 1C), 123.47 (s, 1C), 122.19 (s, 1C), 118.04 (s, 1C), 116.98 (s, 2C). ESI-HRMS: *m*/*z* [M + H]^+^ calcd. for C_21_H_14_Cl_2_NO_4_ 414.0300, found 414.0295.

*(E)-1-(4-(2,4-Dichlorophenoxy)phenyl)-3-(4-nitrophenyl)prop-2-en-1-one* **5t**, orange powder, m.p. 148–150 °C, Yield: 82%. IR (KBr): ʋ_max_ 3075, 1658, 1599, 1577, 1484, 1415, 1415, 1219, 1168, 983, 875, 830, 747, 660, 626 cm^−1^; ^1^H NMR (400 MHz, DMSO-*d*_6_) δ 8.31–8.27 (m, 1H), 8.24 (dt, *J* = 9.1, 2.6 Hz, 2H), 8.16 (dd, *J* = 9.1, 2.3 Hz, 2H), 8.10 (d, *J* = 2.1 Hz, 1H), 7.87–7.76 (m, 2H), 7.53 (ddd, *J* = 8.9, 4.6, 2.4 Hz, 1H), 7.36 (dd, *J* = 8.8, 2.3 Hz, 1H), 7.22–7.12 (m, 1H), 7.10 (dd, *J* = 8.9, 2.2 Hz, 2H); ^13^C NMR (100 MHz, DMSO-*d*_6_) δ 187.94 (s, 1C), 161.13 (s, 1C), 149.61 (s, 1C), 148.52 (s, 1C), 141.62 (s, 1C), 141.41 (s, 1C), 132.84 (s, 1C), 131.94 (s, 2C), 130.91 (s, 1C), 130.52 (s, 1C), 130.32 (s, 2C), 129.73 (s, 1C), 127.16 (s, 1C), 126.37 (s, 1C), 124.52 (s, 1C), 124.42 (s, 2C), 116.99 (s, 2C). ESI-HRMS: *m*/*z* [M + H]^+^ calcd. for C_21_H_14_Cl_2_NO_4_ 414.0300, found 414.0299.

*(E)-3-(4-(4-Chlorophenoxy)phenyl)-1-(4-(2,4-dichlorophenoxy)phenyl)prop-2-en-1-one* **5u**, yellow powder, m.p. 129–131 °C, Yield: 82%. IR (KBr): ʋ_max_ 2980, 1657, 1600, 1580, 1481, 1419, 1293, 1155, 980, 870, 746, 666, 623 cm^−1^; ^1^H NMR (400 MHz, DMSO-*d*_6_) δ 8.19–8.10 (m, 2H), 7.99–7.92 (m, 1H), 7.89 (dp, *J* = 5.9, 2.9 Hz, 2H), 7.76 (dd, *J* = 15.6, 2.4 Hz, 1H), 7.71–7.64 (m, 1H), 7.62–7.55 (m, 2H), 7.46 (dt, *J* = 6.4, 3.9 Hz, 3H), 7.43–7.35 (m, 1H), 7.32–7.21 (m, 1H), 7.21–7.12 (m, 1H), 7.12–7.02 (m, 1H), 6.84 (dd, *J* = 26.0, 7.6 Hz, 1H); ^13^C NMR (100 MHz, DMSO-*d*_6_) δ 188.02 (s, 1C), 160.78 (s, 1C), 158.96 (s, 1C), 155.15 (s, 1C), 143.55 (s, 1C), 133.33 (s, 1C), 131.65 (s, 2C), 131.49 (s, 2C), 130.88 (s, 1C), 130.69 (s, 1C), 130.55 (s, 2C), 129.70 (s, 1C), 128.46 (s, 1C), 127.11 (s, 1C), 124.42 (s, 1C), 122.16 (s, 1C), 121.52 (s, 2C), 121.41 (s, 1C), 118.99 (s, 2C), 118.06 (s, 1C), 116.97 (s, 2C). HRMS (ESI) *m*/*z* calcd for C_27_H_18_Cl_3_O_3_ (M + H)^+^ 495.0322, found 495.0320.

### 3.4. Compounds MIC Testing

The MIC testing of the target compounds was conducted on the following four bacteria stains: *Staphylococcus aureus* (ATCC 25923), *Escherichia coli* (ATCC 25922), *Salmonella* (ATCC 12022), and *Pseudomonas aeruginosa* (ATCC 27853) by microdilution in a 96-well cell culture plate. The 5 μL of suspension was transferred to 5 mL of fresh medium and incubated for a few hours to reach the log phase stage. After that, the bacteria were diluted to reach 2 × 10^6^ colony-forming units per milliliter (CFU mL^−1^). Each well contained 100 μL of the bacterial cell suspension and various concentrations of the target compounds in 100 μL for a final volume of 200 μL wells. The optical density was determined using an enzyme plate analyzer. The experimental results were expressed as OD600, and the experiment was repeated in triplicate.

### 3.5. Constant Concentration Time-Kill Curves

*P. aeruginosa* and *Salmonella* were cultured in an LB liquid medium broth at 37 °C using a shaking incubator at 180 rpm with shaking and then diluted to approximately 6 × 10^5^ CFU mL^−1^. Test compound samples of 3b with final concentrations of 0.5 × MIC, 1 × MIC, 2 × MIC, and 4 × MIC were inoculated with aliquots of bacteria resuspended in fresh media. A small quantity (0.1 mL) was taken from each sample 2, 4, 6, 8, 10, 12, and 24 h following inoculation, and the optical density was determined using an enzyme plate analyzer. The experimental results were expressed as OD600, and the experiment was repeated in triplicate.

### 3.6. Molecular Docking of Compound ***5u*** and Chalcone

Molecular docking studies were performed to investigate the binding mode between the compounds and *P. aeruginosa* LpxC using Autodock vina 1.1.2 [34]. The three-dimensional (3D) structure of the LpxC (PDB ID: 3P3E) was downloaded from RCSB Protein Data Bank (www.rcsb.org). The 3D structures of the compounds were obtained using ChemBioDraw Ultra 14.0 and ChemBio3D Ultra 14.0 software. The AutoDockTools 1.5.6 package was employed to generate the docking input files [36,37]. The search grid of LpxC was identified as center_x: −25.363, center_y: 15.669, and center_z: −5.177 with dimensions size_x: 15, size_y: 15.75, and size_z: 15. The value of exhaustiveness was set to 16. For Vina docking, the default parameters were used if it was not mentioned. The best-scoring pose as judged by the Vina docking score was chosen and visually analyzed using PyMOL 1.7.6 software (www.pymol.org).

### 3.7. Molecular Modeling of Compound ***5u***

#### 3.7.1. Molecular Docking

Molecular docking studies were performed to investigate the binding mode between the compounds and *P*. *aeruginosa* LpxC using Autodock vina 1.1.2 [34]. The three-dimensional (3D) structure of the LpxC (PDB ID: 3P3E) was downloaded from RCSB Protein Data Bank (www.rcsb.org). The 3D structure of the compounds was obtained using ChemBioDraw Ultra 14.0 and ChemBio3D Ultra 14.0 software. The AutoDockTools 1.5.6 package was employed to generate the docking input files [36,37]. The search grid of LpxC was identified as center_x: −25.363, center_y: 15.669, and center_z: −5.177 with dimensions size_x: 15, size_y: 15.75, and size_z: 15. The value of exhaustiveness was set to 16. For Vina docking, the default parameters were used if it was not mentioned. Then, an MD study was performed to revise the docking result.

#### 3.7.2. Molecular Dynamics

The Amber 14 and AmberTools 15 programs were used for MD simulations of the selected docked pose. The compound was first prepared by ACPYPE, a tool based on ANTECHAMBER for generating automatic topologies and parameters in different formats for different molecular mechanics programs, including calculation of partial charges. Then, the forcefield “leaprc.gaff” (generalized amber forcefield) was used to prepare the ligand, while “leaprc.ff14SB” was used for the receptor. The system was placed in a rectangular box (with a 10.0 Å boundary) of TIP3P water using the “SolvateOct” command with the minimum distance between any solute atoms. Equilibration of the solvated complex was achieved by carrying out a short minimization (5000 steps of each steepest descent and conjugate gradient method), 500 ps of heating, and 50 ps of density equilibration with weak restraints using the GPU (NVIDIA^®^ Tesla K20c, NVIDA, Santa Clara, CA, USA) accelerated PMEMD (Particle Mesh Ewald Molecular Dynamics) module. Finally, 40 ns of MD simulations were carried out. All the molecular dynamics were performed on a Dell Precision T5500 workstation (Dell, Austin, TX, USA).

#### 3.7.3. Binding Free Energy and Energy Decomposition per Residue Calculations

The binding free energies (ΔG_bind_ in kcal/mol) were calculated using the molecular mechanics/generalized born surface area (MM/GBSA) method, implemented in AmberTools 15. Moreover, to identify the key protein residues responsible for the ligands binding process, the binding free energy was decomposed on a per-residue basis. For each complex, the binding free energy of MM/GBSA was estimated as follows:

ΔG_bind_= G_complex_ − G_protein_ − G_ligand_

where ΔG_bind_ is the binding free energy and G_complex_, G_protein_, and G_ligand_ are the free energies of complex, protein, and ligand, respectively.

## 4. Conclusions

In conclusion, a novel series of chalcone derivatives, **5a**-**5u**, incorporating a diphenyl ether moiety, was designed in pursuit of innovative antibacterial agents. The newly synthesized molecules were prepared and characterized by NMR, HR-MS, and FR-IR techniques. The biological evaluation of these compounds was conducted against both Gram-negative and Gram-positive bacteria. In vitro results demonstrated that most of the target compounds exhibited significant potency in inhibiting bacterial growth. In particular, compound **5u** with two diphenyl ether moieties displayed exceptional antibacterial activity. Molecular modeling of the *P. aeruginosa* LpxC-compound **5u** complex suggested that compound **5u** could strongly bind to and interact with the binding site of the LpxC. Based on these findings, compound **5u** represents a promising lead for further optimization and development.

## Figures and Tables

**Table 1:** Lipinski’s rule of five parameters of the synthesized chalcone derivatives **5a**-**5u**.

Compd	R	MW	logP	HBA	HBD
**5a**	2-F	387.23	4.82	3	0
**5b**	2-Cl	403.68	5.66	2	0
**5c**	2-Br	448.14	5.60	2	0
**5d**	2-CH_3_	383.27	4.73	2	0
**5e**	2-CH_3_O	399.27	4.40	3	0
**5f**	2-CF_3_	437.24	5.32	2	0
**5g**	3-F	387.23	4.82	3	0
**5h**	3-Cl	403.68	5.66	2	0
**5i**	3-Br	448.14	5.60	2	0
**5j**	3-CH_3_	383.27	5.01	2	0
**5k**	3-CH_3_O	399.27	4.40	3	0
**5l**	3-CF_3_	437.24	5.32	2	0
**5m**	4-F	387.23	4.82	3	0
**5n**	4-Cl	403.68	5.66	2	0
**5o**	4-Br	448.14	5.60	2	0
**5p**	4-CH_3_	383.27	4.73	2	0
**5q**	4-CH_3_O	399.27	4.40	3	0
**5r**	4-CF_3_	437.24	5.32	2	0
**5s**	3-NO_2_	414.24	4.20	4	0
**5t**	4-NO_2_	414.24	4.18	6	0
**5u**	4-(4-ClPh)O	495.78	6.82	3	0

MW: Molecular weight; logP: logarithm of octanol–water partition coefficient; HBA: number of hydrogen bond acceptors; HBD: number of hydrogen bond donors.

**Table 2:** Antibacterial activity of the synthesized chalcone derivatives **5a**-**5u**.

Compd	MIC (μM)			
	*S. aureus*	*E. coli*	*Salmonella*	*P. aeruginosa*
**5a**	68.85	45.09	45.09	45.09
**5b**	58.65	39.10	39.10	39.10
**5c**	33.48	33.48	33.48	33.48
**5d**	34.80	34.80	34.80	34.80
**5e**	66.80	33.40	33.40	33.40
**5f**	76.25	50.83	50.83	50.83
**5g**	68.85	45.90	45.90	45.90
**5h**	44.60	29.73	29.73	29.73
**5i**	55.05	27.53	36.70	27.53
**5j**	66.10	44.07	44.07	44.07
**5k**	83.50	33.40	33.40	33.40
**5l**	76.25	38.13	38.13	38.13
**5m**	71.73	71.73	71.73	71.73
**5n**	71.00	47.33	47.33	47.33
**5o**	67.25	44.83	44.83	44.83
**5p**	77.40	51.60	51.60	51.60
**5q**	66.80	44.53	44.53	44.53
**5r**	50.83	76.25	50.83	50.83
**5s**	60.35	40.23	40.23	40.23
**5t**	64.40	42.93	42.93	32.20
**5u**	25.23	33.63	33.63	33.63
chalcone	80.03	106.70	106.70	106.70
ciprofloxacin	9.72	0.78	259.17	388.75
gentamycin	6.51	1.04	52.11	521.10

## Data Availability

Data are contained within the article and Supplementary Materials.

## Supplementary Materials

The following supporting information can be downloaded at https://www.mdpi.com/article/10.3390/molecules30122575/s1, Compound spectra.

## References

[1] George S., Muhaj F.F., Nguyen C.D., Tyring S.K. (2022). Part I antimicrobial resistance: Bacterial pathogens of dermatologic significance and implications of rising resistance. J. Am. Acad. Dermatol..

[2] Falkow S. (2006). Is persistent bacterial infection good for your health?. Cell.

[3] Shanbhag C., Saraogi I., Bacterial G.T. (2023). Pases as druggable targets to tackle antimicrobial resistance. Bioorg. Med. Chem. Lett..

[4] Ao S., Linke L., Magnuson R., Jauch L., Hyatt D.R. (2022). Antimicrobial resistance and genetic diversity of *Staphylococcus aureus* collected from livestock, poultry and humans. One Health.

[5] Chahouri A., Radouane N., Yacoubi B., Moukrim A., Banaoui A. (2022). Microbiological assessment of marine and estuarine ecosystems using fecal indicator bacteria, Salmonella, Vibrio and antibiotic resistance pattern. Mar. Pollut. Bull..

[6] González-Bello C. (2017). Antibiotic adjuvants-A strategy to unlock bacterial resistance to antibiotics. Bioorg. Med. Chem. Lett..

[7] GBD 2021 Antimicrobial Resistance Collaborators (2024). Global burden of bacterial antimicrobial resistance 1990–2021: A systematic analysis with forecasts to 2050. Lancet.

[8] Jin L., Zhang X., Luo Z., Wu X., Zhao Z. (2023). Synthesis and antibacterial activity of novel 2-fluoro ketolide antibiotics with 11,12-quinoylalkyl side chains. Bioorg. Med. Chem. Lett..

[9] El-Barasi N.M., Miloud M.M., El-ajaily M.M., Mohapatra R.K., Sarangi A., Das D., Mahal A., Parhi P.K., Pintilie L., Barik S.R. (2020). Synthesis, structural investigations and antimicrobial studies of hydrazone based ternary complexes with Cr(III), Fe(III) and La(III) ions. J. Saudi Chem. Soc..

[10] Duan M., Mahal A., Mohammed B., Zhu Y., Tao H., Mai S., Al-Haideri M., Zhu Q. (2022). Synthesis and antitumor activity of new tetrahydrocurcumin derivatives via click reaction. Nat. Prod. Res..

[11] Zinad D.S., Mahal A., Mohapatra R.K., Sarangi A.K., Pratama M.R.F. (2020). Medicinal chemistry of oxazines as promising agents in drug discovery. Chem. Biol. Drug Des..

[12] WHO (2023). World AMR Awareness Week: Preventing Antimicrobial Resistance Together.

[13] Zhuang C.L., Zhang W., Sheng C.Q., Zhang W.N., Xing C.G., Miao Z.Y. (2017). Chalcone: A privileged structure in medicinal chemistry. Chem. Rev..

[14] Chen J., Li Y., Yang L.Q., Li Y.Z., Nan Z.B., Gao K. (2012). Biological activities of flavonoids from pathogenic-infected Astragalus adsurgens. Food Chem..

[15] Dan W.J., Dai J.K. (2020). Recent developments of chalcones as potential antibacterial agents in medicinal chemistry. Eur. J. Med. Chem..

[16] Zhao X., Mei W., Gong M., Zuo W., Bai H., Dai H. (2011). Antibacterialactivity of the flavonoids from Dalbergia odorifera on Ralstoniasolanacearum. Molecules.

[17] Sufian A.S., Ramasamy K., Ahmat N., Zakaria Z.A., Yusof M.I.M. (2013). Isolation and identification of antibacterial and cytotoxic compounds from the leaves of *Muntingia calabura* L.. J. Ethnopharmacol..

[18] Mahapatra D.K., Bharti S.K., Asati V., Singh S.K. (2019). Perspectives of medicinally privileged chalcone based metal coordination compounds for biomedical applications. Eur. J. Med. Chem..

[19] Matos M.J., Vazquez-Rodriguez S., Uriarte E., Santana L. (2005). Potential pharmacological uses of chalcones: A patent review (from June 2011–2014). Expert. Opin. Ther. Pat..

[20] Sahu N.K., Balbhadra S.S., Choudhary J., Kohli D.V. (2012). Exploring pharmacological significance of chalcone scaffold: A review. Curr. Med. Chem..

[21] Nowakowska Z. (2007). A review of anti-infective and anti-inflammatory chalcones. Eur. J. Med. Chem..

[22] Xin Y., Jin H., Hou R.T., Chen L., Chen F.L., Wu K.Q., Wang Y.L., Yang Z.R. (2006). Synthesis and antimicrobial activities of brominated dihydroxy diphenyl ethers. Chem. Res. Appl..

[23] Jin H., Chen S., Hou R.T., Wang Y.L., Chen S.H., Yang Z.R. (2006). Study on the synthesis and antimicrobial activities of brominated dihydroxy diphenyl ethers. Chin. J. Org. Chem..

[24] Jin H., Zhou J., Pu T., Zhang A., Gao X., Tao K., Hou T. (2017). Synthesis of novel fenfuram-diarylether hybrids as potent succinate dehydrogenase inhibitors. Bioorg. Chem..

[25] Wen F., Jin H., Tao K., Hou T.P. (2016). Design, synthesis and antifungal activity of novel furancarboxamide derivatives. Eur. J. Med. Chem..

[26] Zhu J.J., Huang Q.S., Liu S.Q., Ding W.J., Xiong Y.H., Li C.Y. (2022). Four new diphenyl ether derivatives from a mangrove endophytic fungus *Epicoccum sorghinum*. Chin. J. Nat. Med..

[27] Chen T., Xiong H., Yang J.F., Zhu X.L., Qu R.Y., Yang G.F. (2020). Diaryl ether: A privileged scaffold for drug and agrochemical discovery. J. Agric. Food Chem..

[28] Zhu J., Li Z., Lu H., Liu S., Ding W., Li J., Xiong Y., Li C. (2021). New diphenyl ethers from a fungus *Epicoccum sorghinum* L28 and their antifungal activity against phytopathogens. Bioorg. Chem..

[29] Jin H., Geng Y.C., Yu Z.Y., Tao K., Hou T.P. (2009). Lead optimization and anti-plant pathogenic fungi activities of daphneolone analogues from *Stellera chamaejasme* L.. Pestic. Biochem. Phys..

[30] Liu W., Shi H.M., Jin H., Zhao H.Y., Zhou G.P., Wen F., Yu Z.Y., Hou T.P. (2009). Design, synthesis and antifungal activity of a series of novel analogs based on diphenyl ketones. Chem. Biol. Drug Des..

[31] Zhang H., Jin H., Ji L.Z., Tao K., Liu W., Zhao H.Y., Hou T.P. (2011). Design, synthesis, and bioactivities screening of a diaryl ketone-inspired pesticide molecular library as derived from natural products. Chem. Biol. Drug Des..

[32] Yang Q.Q., Liu N., Yan J.Y., Ren Z.L., Wang L. (2020). Visible light- and heat-promoted C−O coupling reaction of phenols and aryl halides. Asian. J. Org. Chem..

[33] Walsh S., Severino A., Zhou C.Y., He J.F., Liang G.B., Tan C.P., Cao J., Eiermann G.J., Xu L., Salituro G. (2011). 3-Substituted 3-(4-aryloxyaryl)-propanoic acids as GPR40 agonists. Bioorg. Med. Chem. Lett..

[34] Li S.Y., Jin H. (2025). Preparation of Diphenyl Ether Chalcone Derivatives and Its Application. China patent.

[35] Kozłowska J., Potaniec B., Baczyńska D., Żarowska B., Anioł M. (2019). Synthesis and biological evaluation of novel aminochalcones as potential anticancer and antimicrobial agents. Molecules.

[36] Pei S., Lai L., Sun W., Lu Z., Hao J., Liu Y., Wu W., Guan S., Su X. (2024). Discovery of novel tetrahydrobenzothiophene derivatives as, M.S.;BA inhibitors for antimicrobial agents. Bioorg. Chem..

[37] Liu X.W., Yang Y.J., Zhe Qin Z., Li S.H., Bai L.X., Ge W.B., Li J.Y. (2022). Isobavachalcone from cullen corylifolium presents significant antibacterial activity against clostridium difficile through disruption of the cell membrane. Front. Pharmacol..

[38] Okolo E., Ugwu D.I., Ezema B.E., Ndefo J.C., Eze F.U., Ezema C.G., Ezugwu J.A., Ujam O. (2021). New chalcone derivatives as potential antimicrobial and antioxidant agent. Sci. Rep..

[39] Trott O., Olson A.J. (2010). AutoDock Vina: Improving the speed and accuracy of docking with a new scoring function, efficient optimization, and multithreading. J. Comput. Chem..

[40] Sanner M.F. (1999). Python: A programming language for software integration and development. J. Mol. Graph Model..

[41] Morris G.M., Huey R., Lindstrom W., Sanner M.F., Belew R.K., Goodsell D.S., Olson A.J. (2009). AutoDock4 and AutoDockTools 4: Automated docking with selective receptor flexibility. J. Comput. Chem..

[42] Pierce L.C., Salomon-Ferrer R., Augusto F., de Oliveira C., McCammon J.A., Walker R.C. (2012). Routine access to millisecond time scale events with accelerated molecular dynamics. J. Chem. Theory Comput..

[43] Götz A.W., Williamson M.J., Xu D., Poole D., Le Grand S., Walker R.C. (2012). Routine microsecond molecular dynamics simulations with AMBER on GPUs. 1. Generalized Born. J. Chem. Theory Comput..

[44] Salomon-Ferrer R., Götz A.W., Poole D., Le Grand S., Walker R.C. (2013). Routine microsecond molecular dynamics simulations with Amber on GPUs. 2. Explicit solvent particle mesh Ewald. J. Chem. Theory Comput..

[45] Da Silva A.W.S., Vranken W.F. (2012). ACPYPE-Antechamber python parser interface. BMC Res. Notes.

[46] Wang J., Wolf R.M., Caldwell J.W., Kollman P.A., Case D.A. (2004). Development and testing of a general amber force field. J. Comput. Chem..

[47] Wang J., Wang W., Kollman P.A., Case D.A. (2006). Automatic atom type and bond type perception in molecular mechanical calculations. J. Mol. Graph Model..

[48] Cheng T., Zhao Y., Li X., Lin F., Xu Y., Zhang X.L., Li Y., Wang R.X. (2007). Computation of octanol-water partition coefficients by guiding an additive model with knowledge. J. Chem. Inf. Model..

