# Supplementary material for: Synthesis, Antibacterial Evaluation and Molecular Modeling of Novel Chalcone Derivatives Incorporating the Diphenyl Ether Moiety
Source: Molecules. 2025 Jun 13;30(12):2575. doi: 10.3390/molecules30122575 (PMC12196190; doi:10.3390/molecules30122575)

Supplementary Data

Synthesis, Antibacterial Evaluation and Molecular Modeling of Novel Chalcone Derivatives Incorporating the Diphenyl Ether Moiety

Shiyuan Li [1], Hong Jin [2,*]

[1] *Key Laboratory of Drug-Targeting and Drug Delivery System, West China School of Pharmacy, Sichuan University, Chengdu, 610041, China*

[2] *Key Laboratory of Bio-Resource and Eco-Environment of Ministry of Education, College of Life Sciences, Sichuan University, Chengdu, 610065, China*

*Corresponding jinhong@scu.edu.cn (H. Jin).

1. Compound spectra

The ^{1}H NMR spectrum of compound **5a**

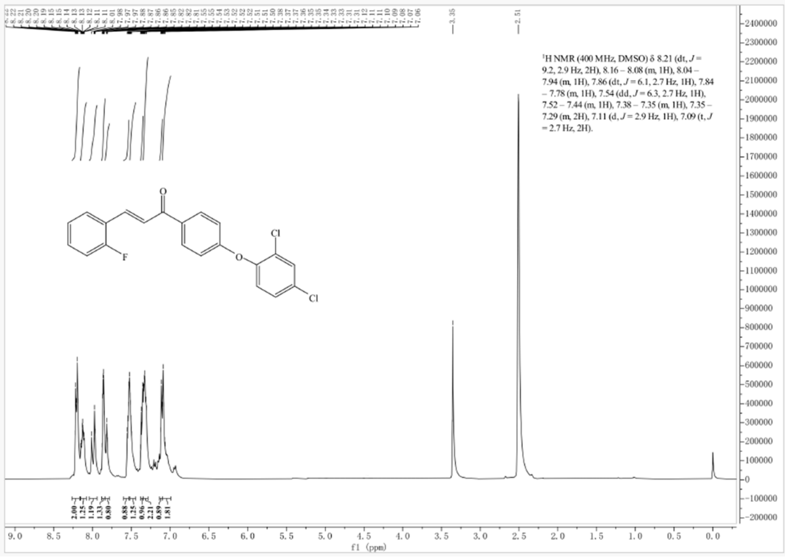

The ^{13} C NMR spectrum of compound **5a**

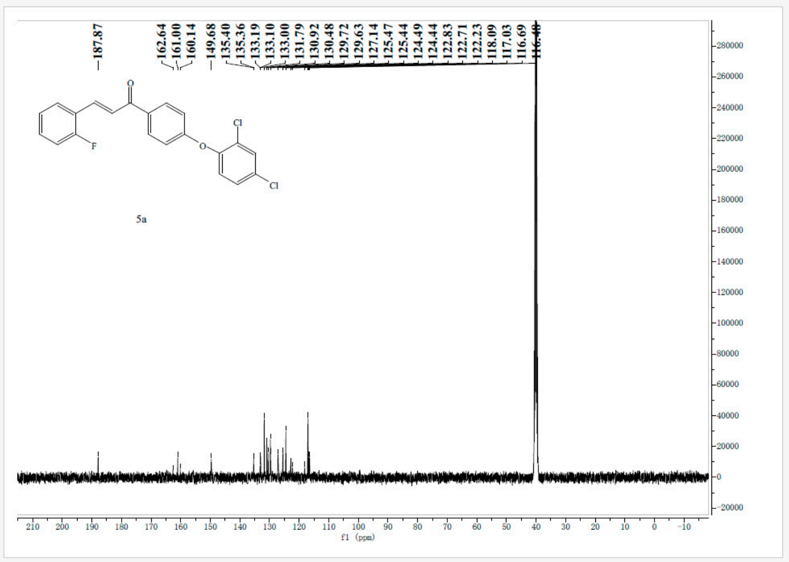

The IR spectrum of compound **5a**

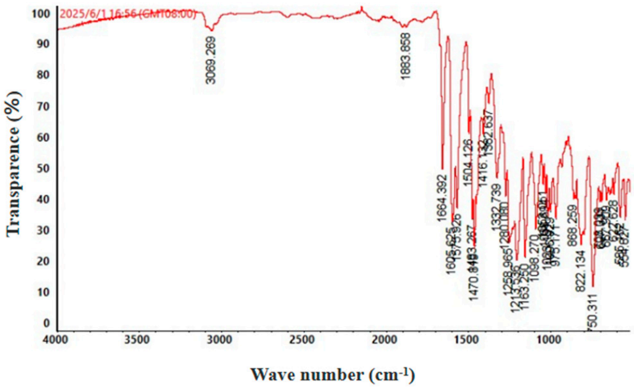

The ^{1}H NMR spectrum of compound **5b**

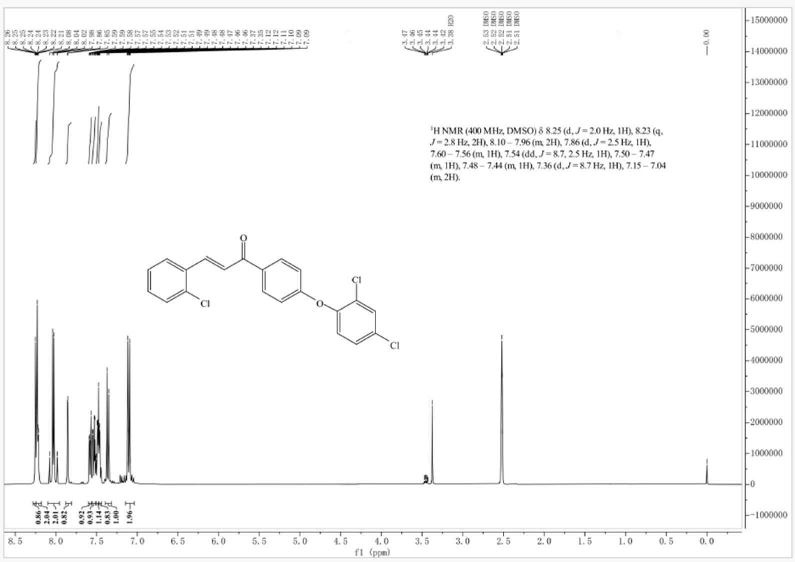

The ^{13} C NMR spectrum of compound **5b**

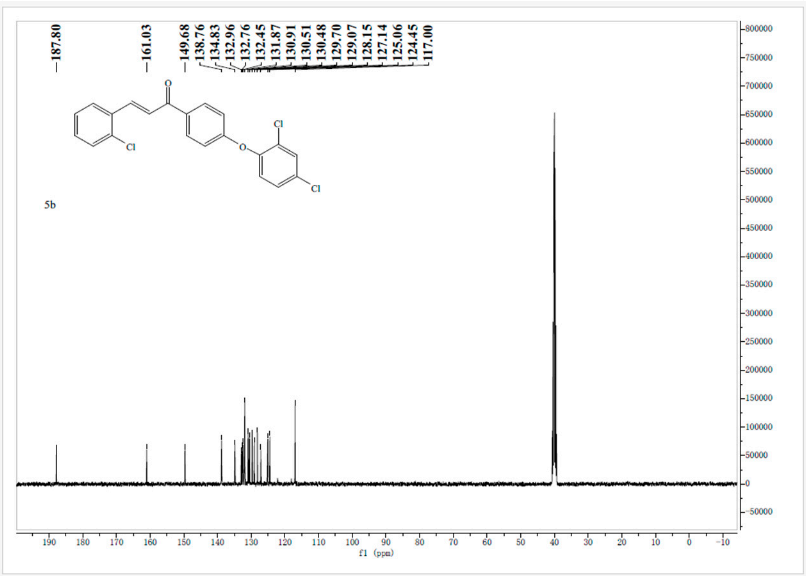

The IR spectrum of compound **5b**

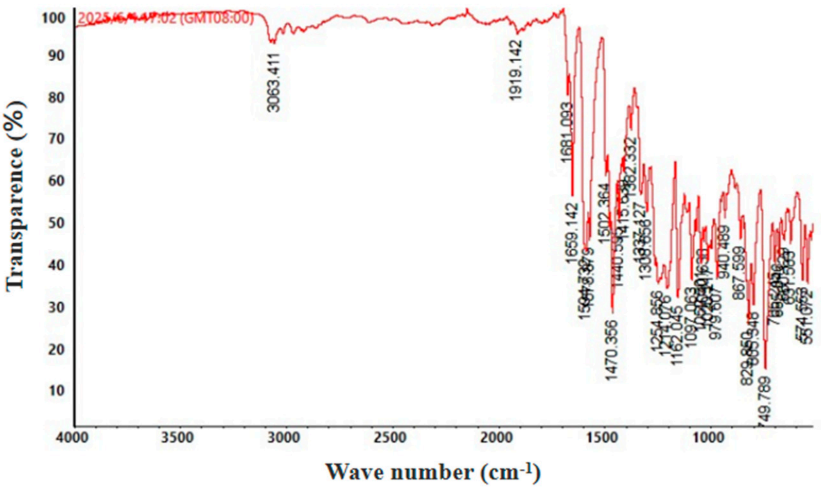

The ^{1}H NMR spectrum of compound **5c**

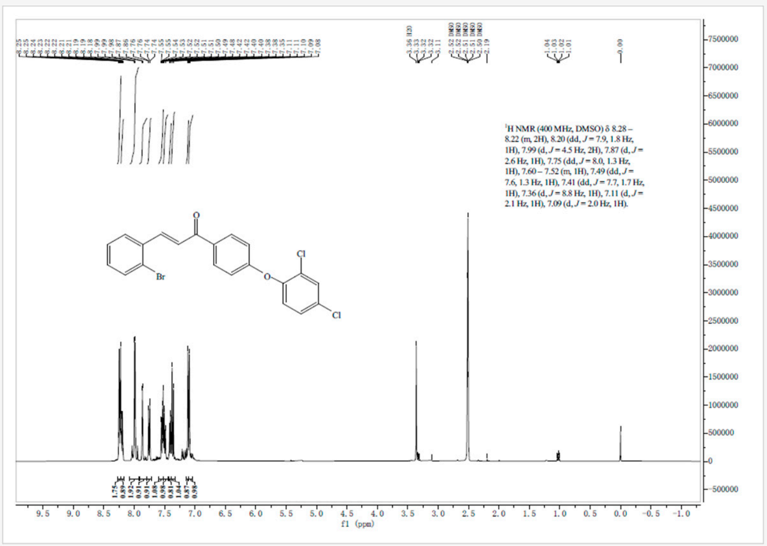

The ^{13} C NMR spectrum of compound **5c**

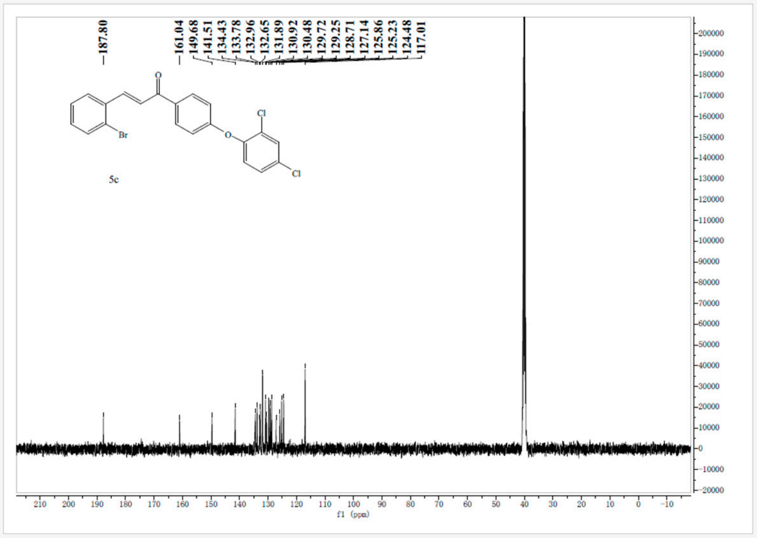

The IR spectrum of compound **5c**

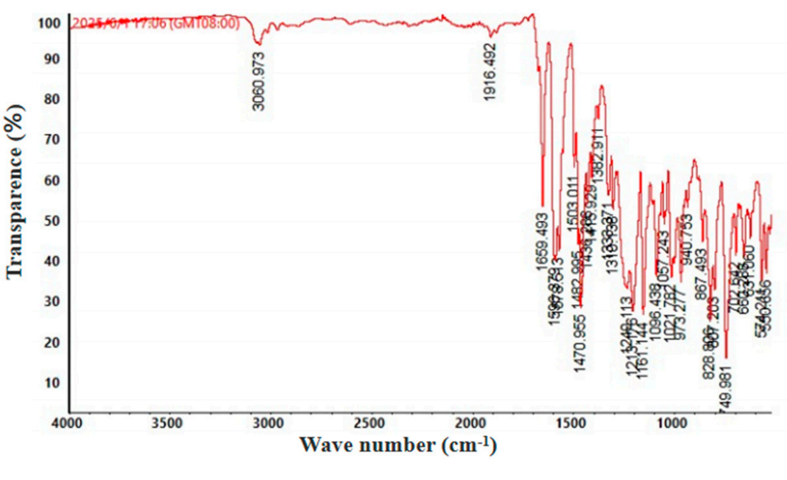

The ^{1}H NMR spectrum of compound **5e**

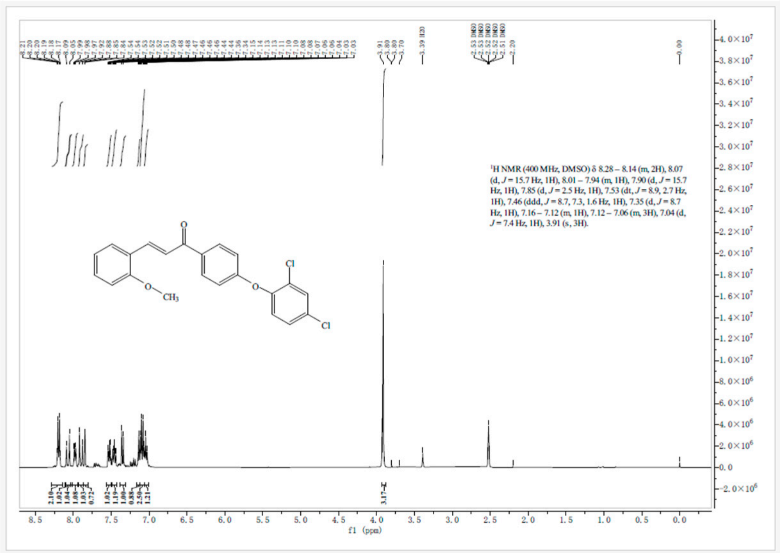

The ^{13}C NMR spectrum of compound **5e**

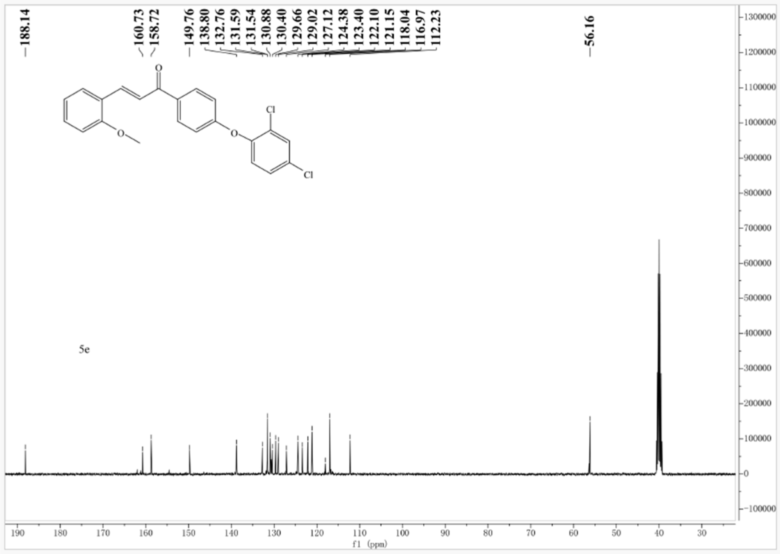

The IR spectrum of compound **5e**

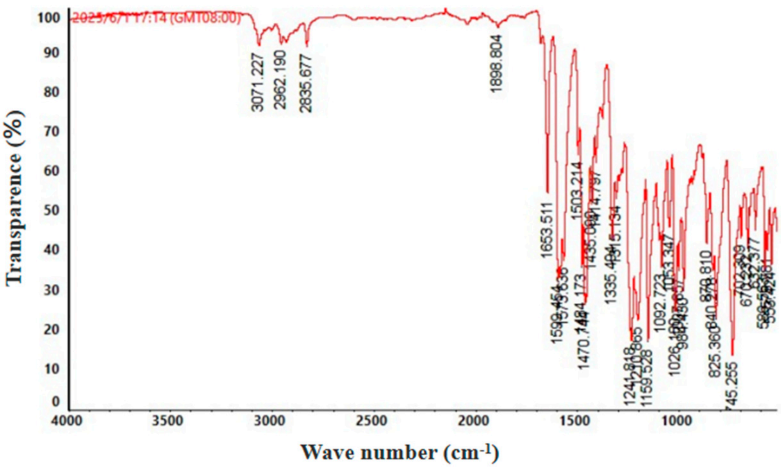

The ^{1}H NMR spectrum of compound **5f**

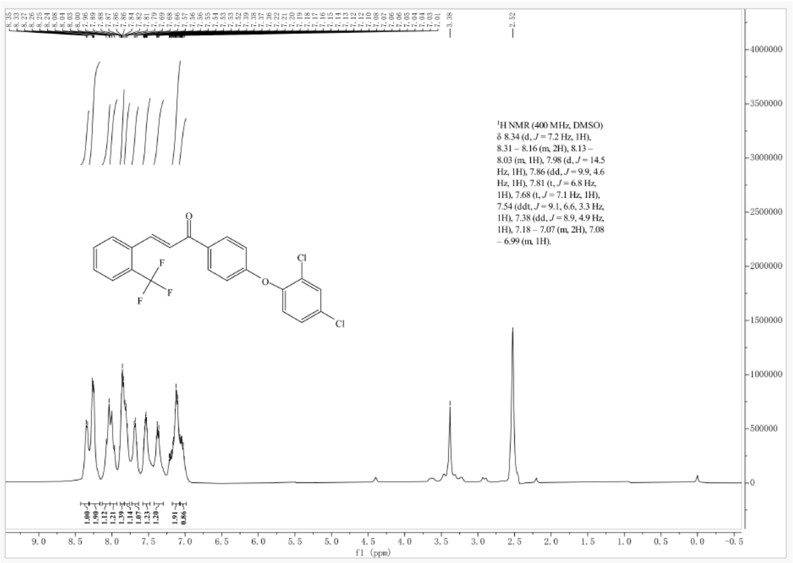

The ^{13} C NMR spectrum of compound **5f**

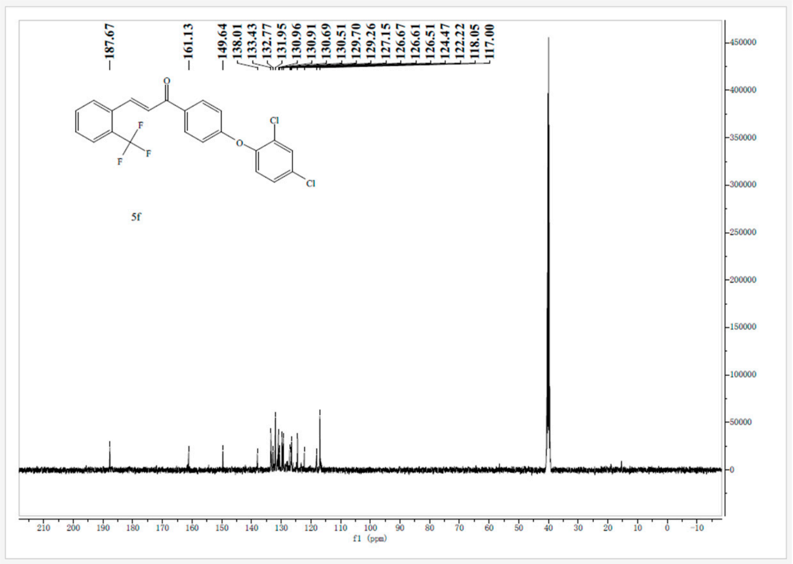

The IR spectrum of compound **5f**

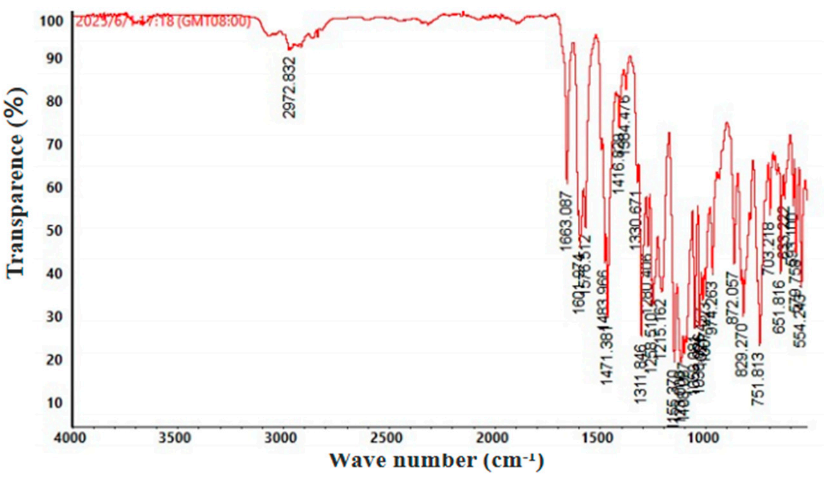

The ^{1}H NMR spectrum of compound **5g**

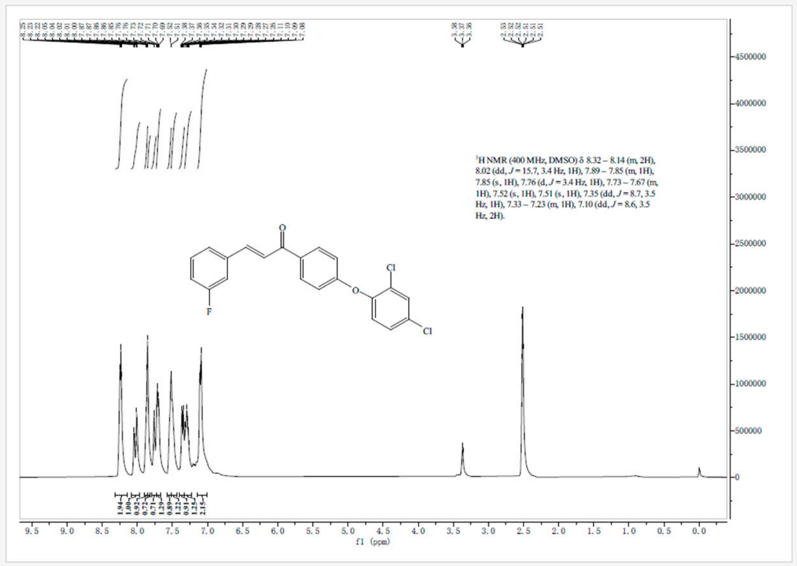

The ^{13}C NMR spectrum of compound **5g**

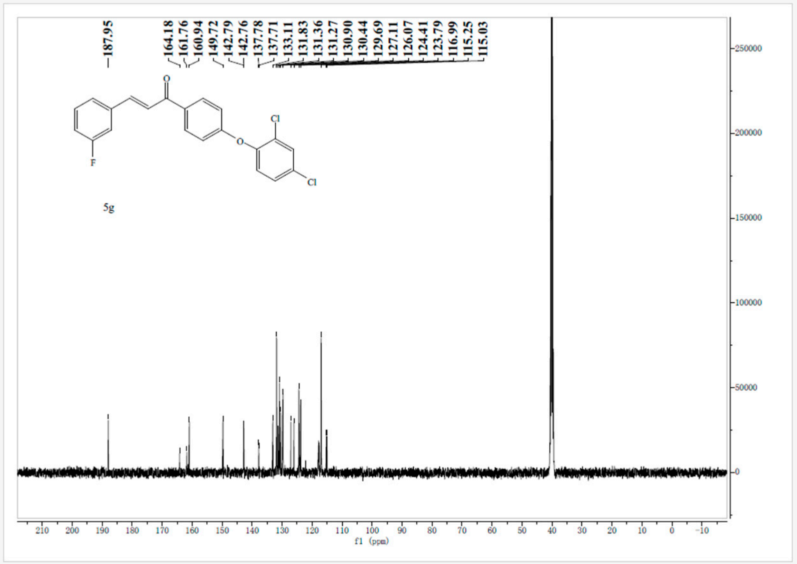

The IR spectrum of compound **5g**

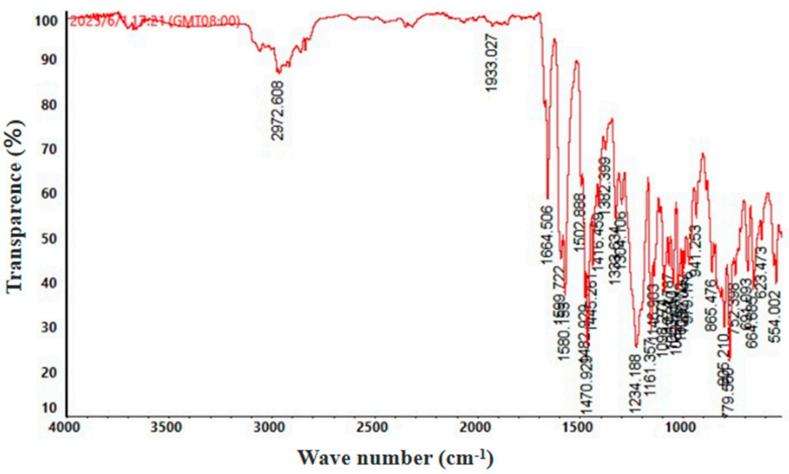

The ^{1}H NMR spectrum of compound **5h**

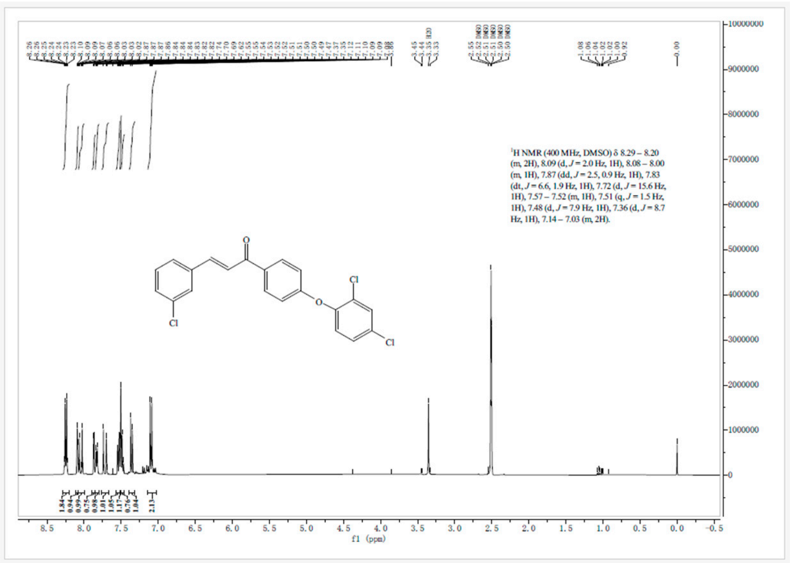

The ^{13} C NMR spectrum of compound **5h**

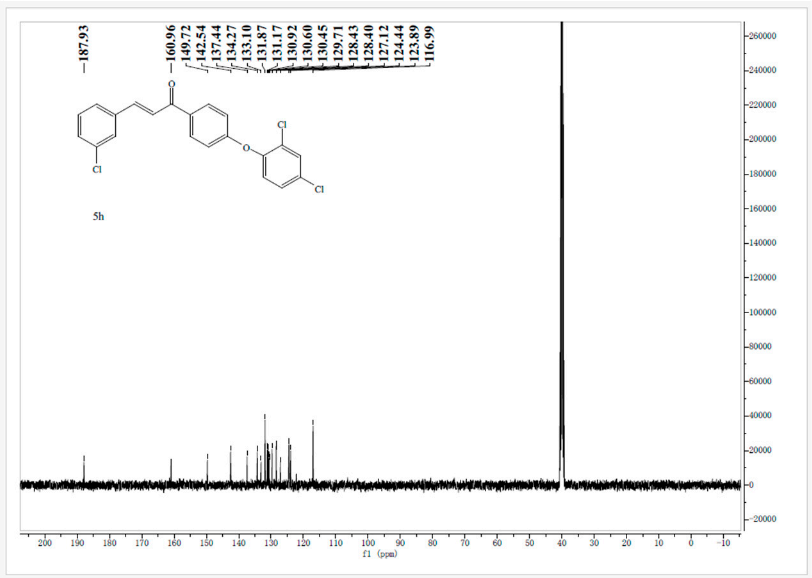

The IR spectrum of compound **5h**

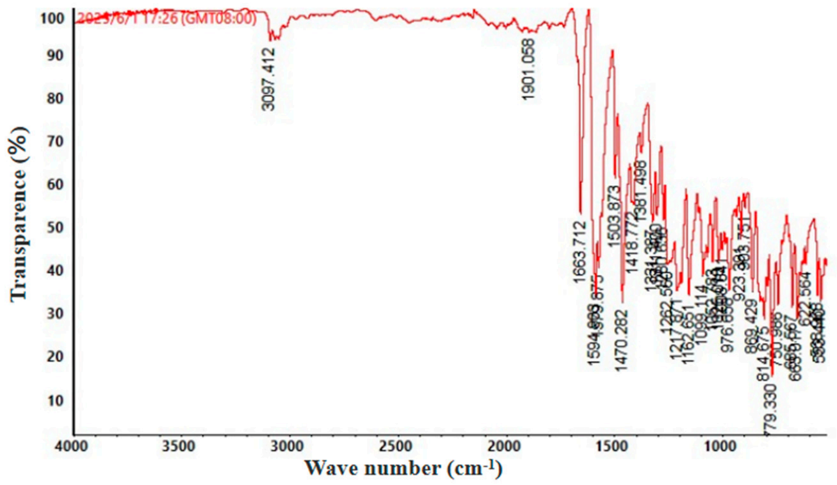

The ^{1}H NMR spectrum of compound **5i**

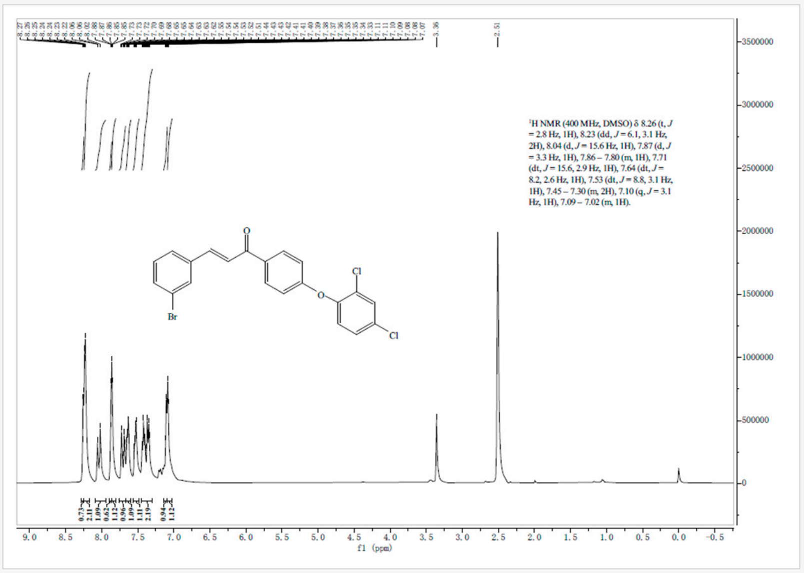

The [13] C NMR spectrum of compound **5i**

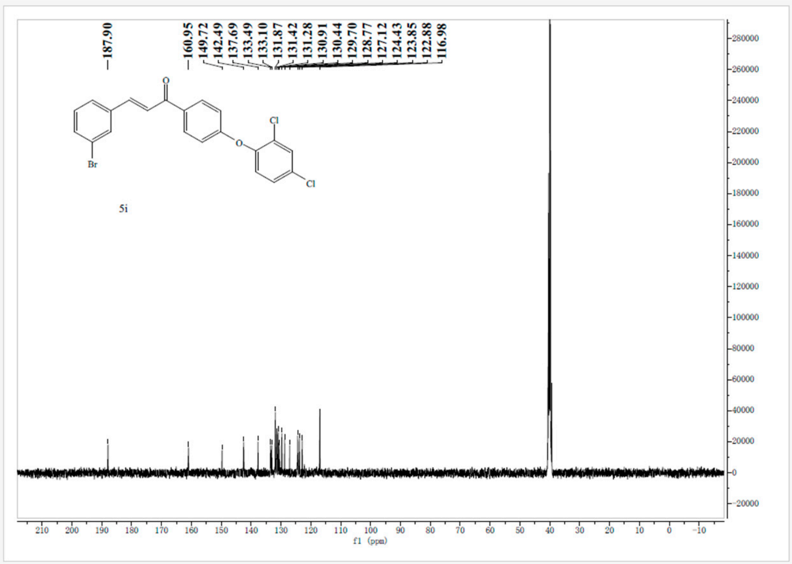

The IR spectrum of compound **5i**

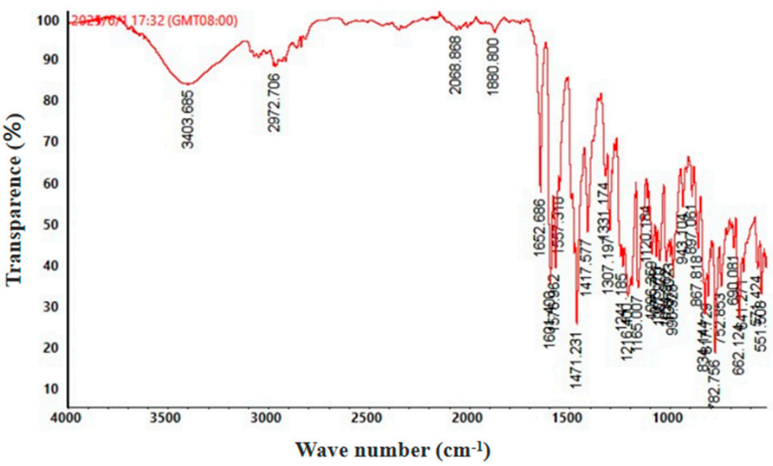

The ^{1}H NMR spectrum of compound **5j**

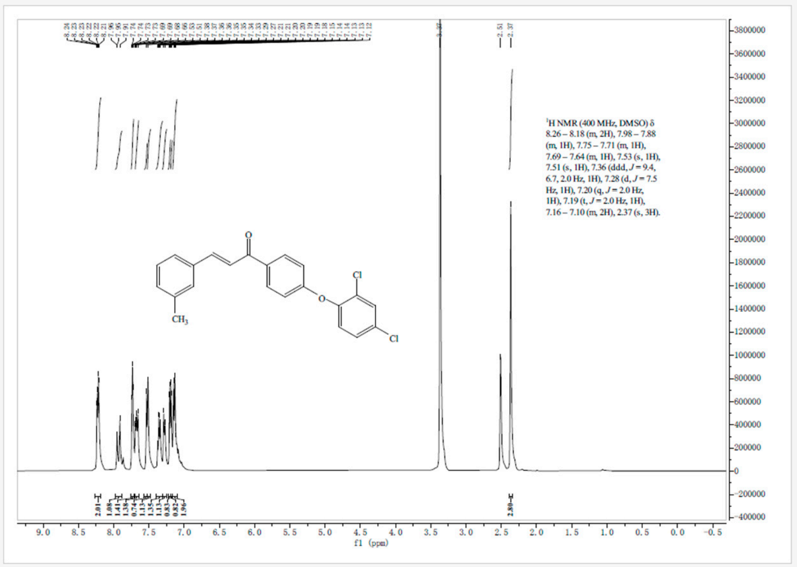

The [13] C NMR spectrum of compound **5j**

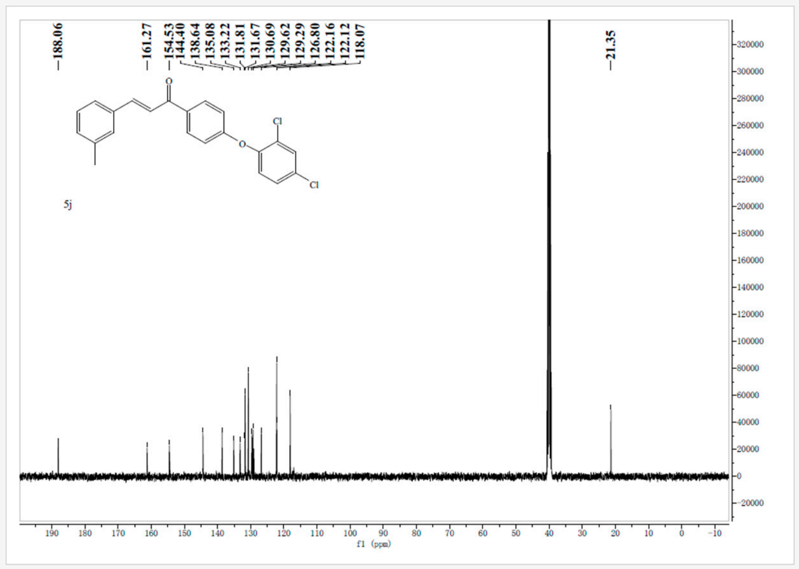

The IR spectrum of compound **5j**

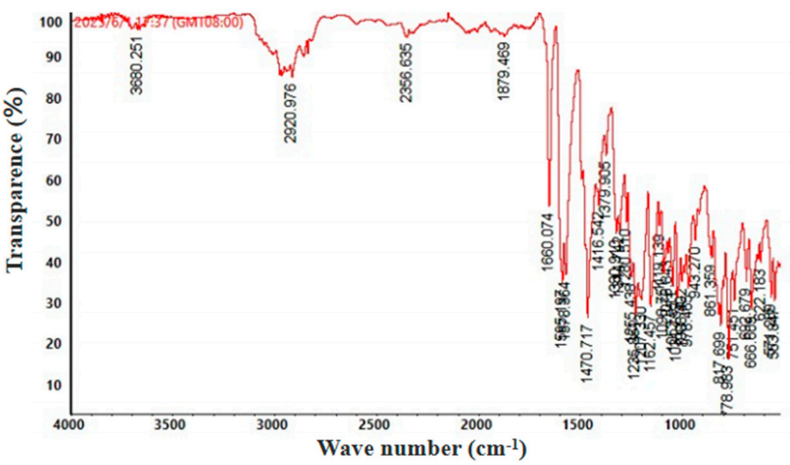

The ^{1}H NMR spectrum of compound **5k**

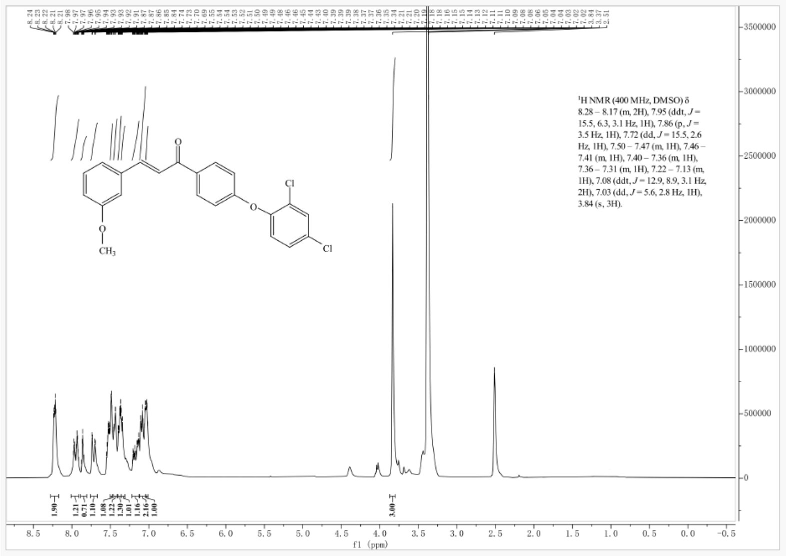

The [13] C NMR spectrum of compound **5k**

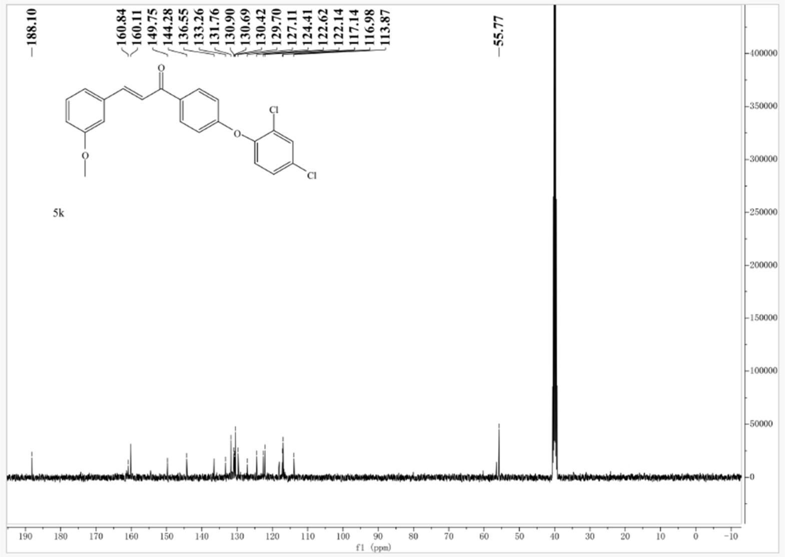

The IR spectrum of compound **5k**

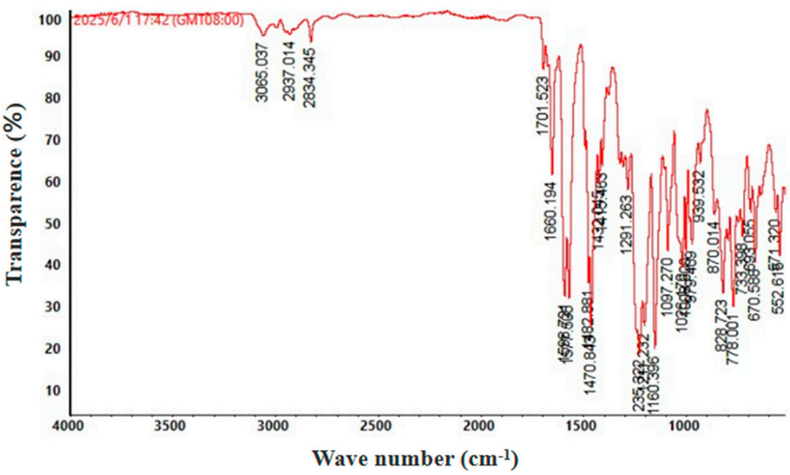

The ^{1}H NMR spectrum of compound **5l**

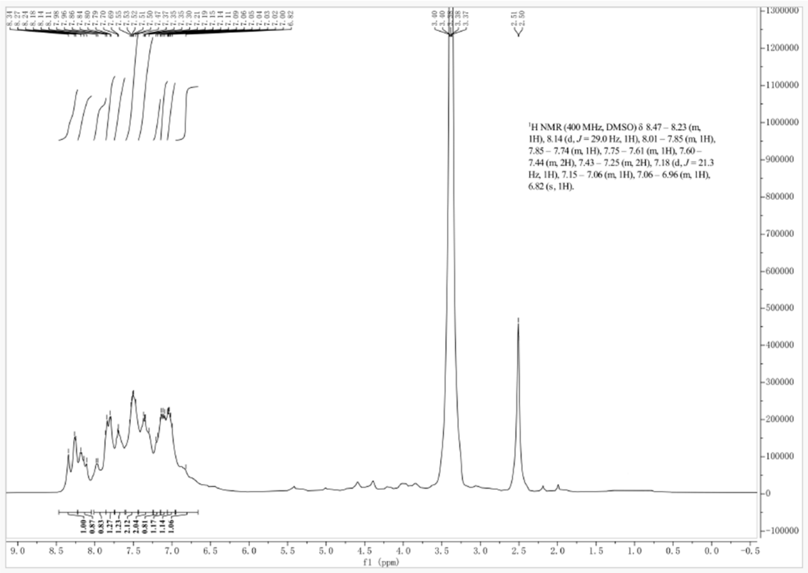

The ^{13} C NMR spectrum of compound **5l**

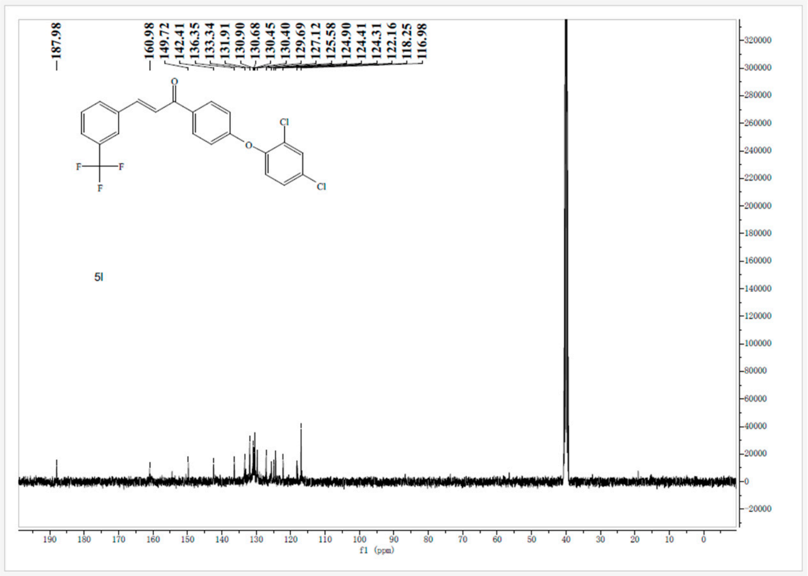

The IR spectrum of compound **5l**

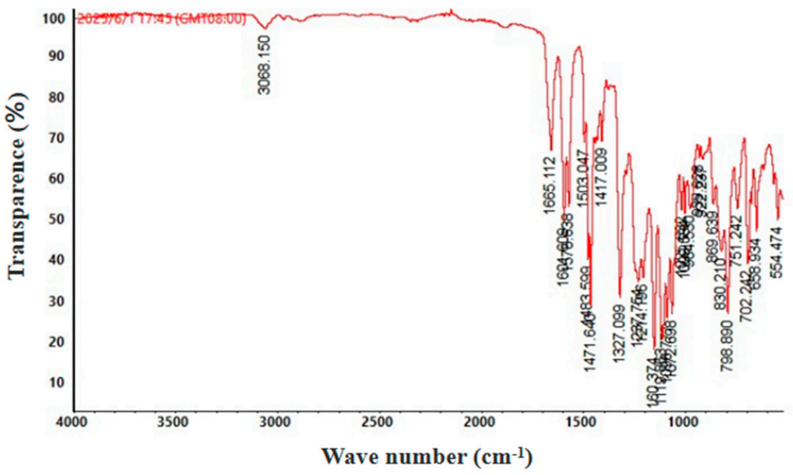

The ^{1}H NMR spectrum of compound **5m**

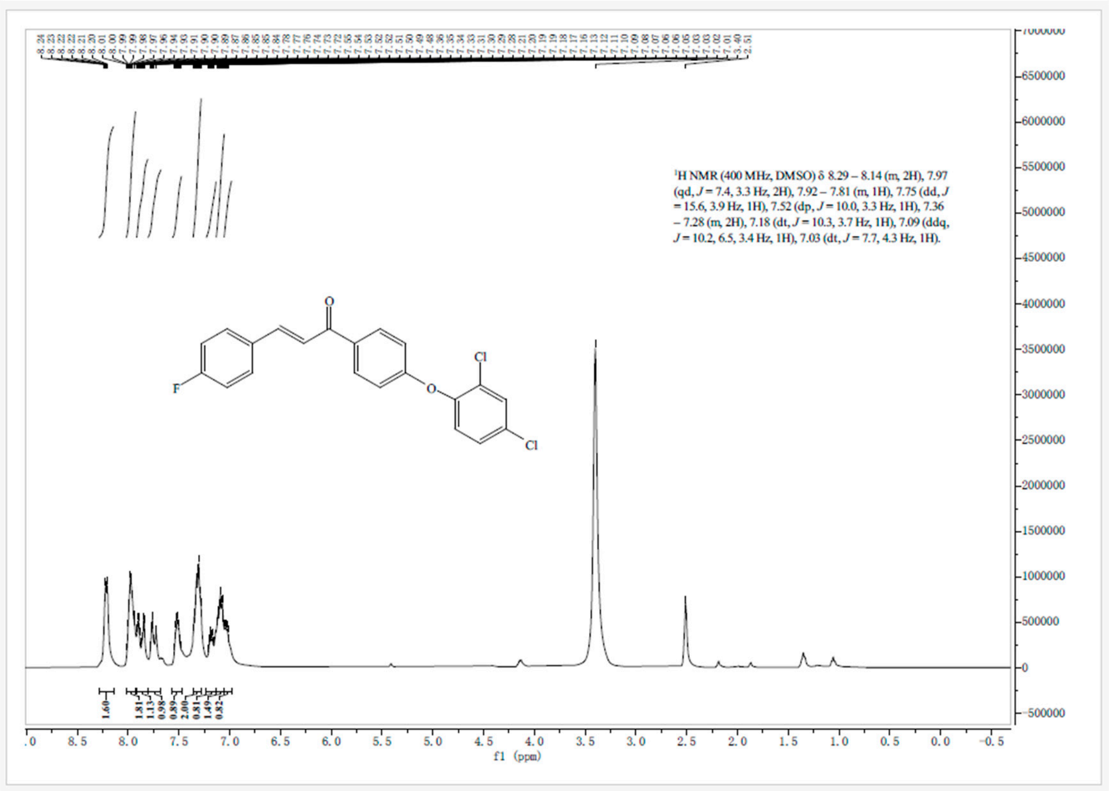

The [13] C NMR spectrum of compound **5m**

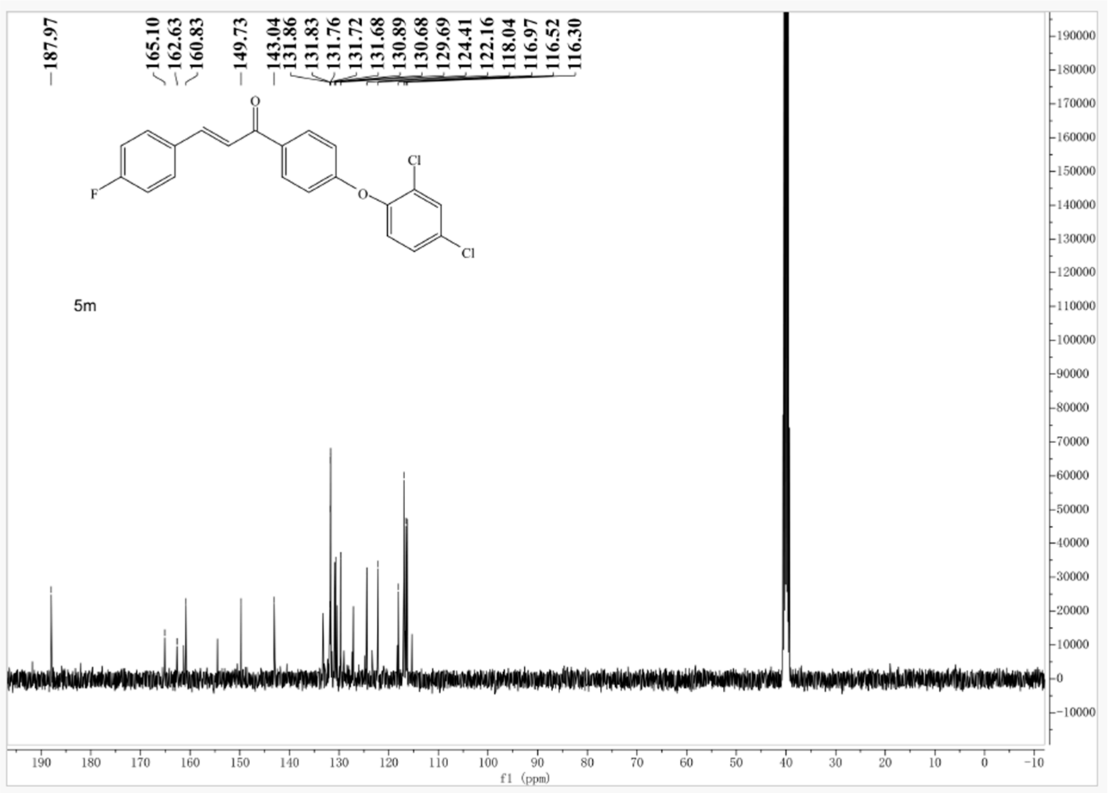

The HRMS of compound **5m**

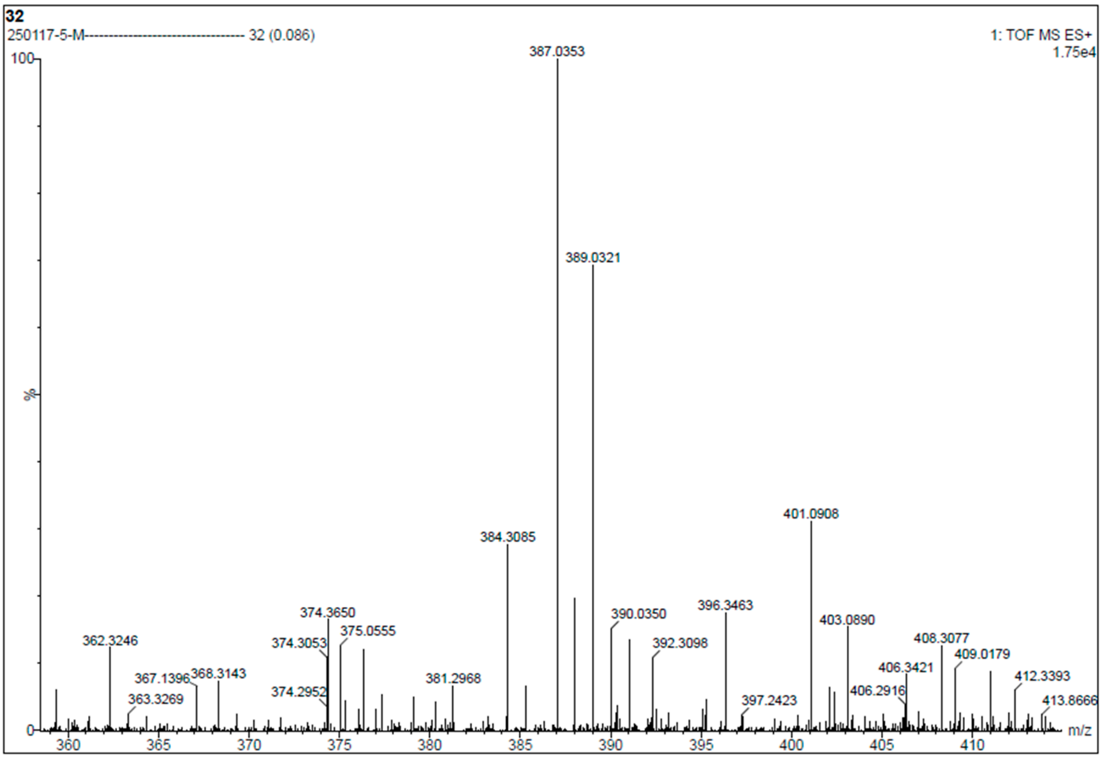

The IR of compound **5m**

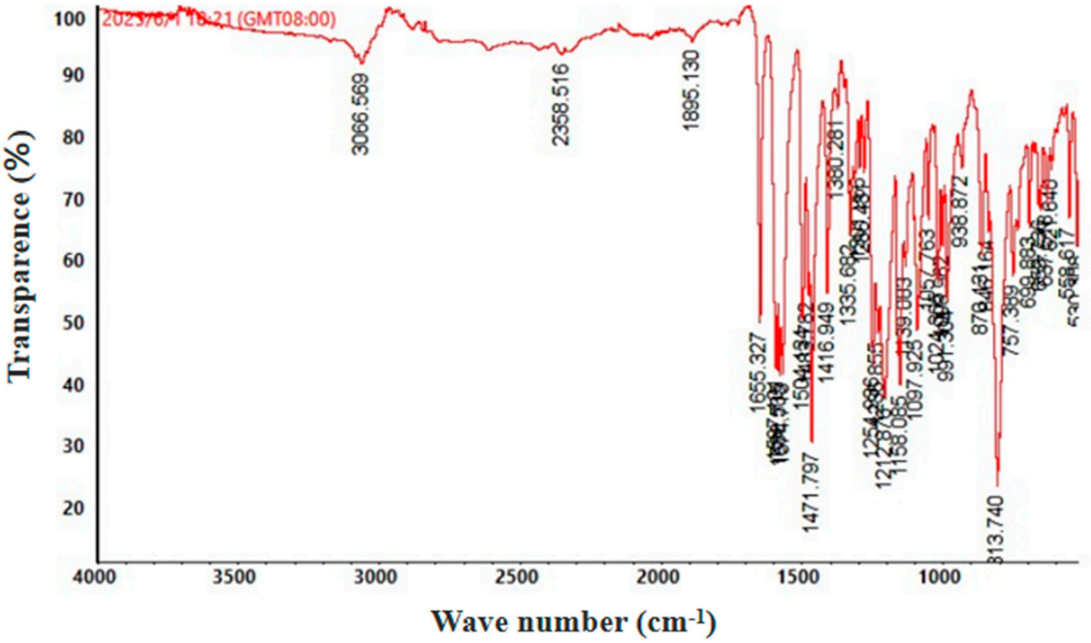

The ^{1}H NMR spectrum of compound **5o**

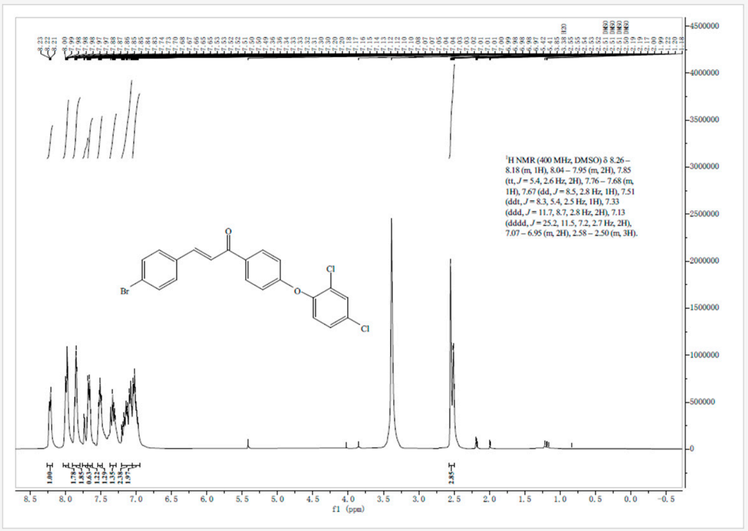

The [13] C NMR spectrum of compound **5o**

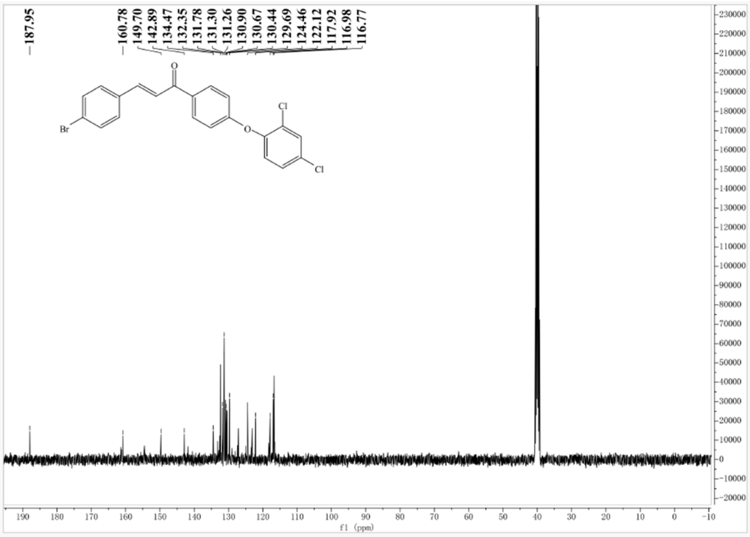

The HRMS of compound **5o**

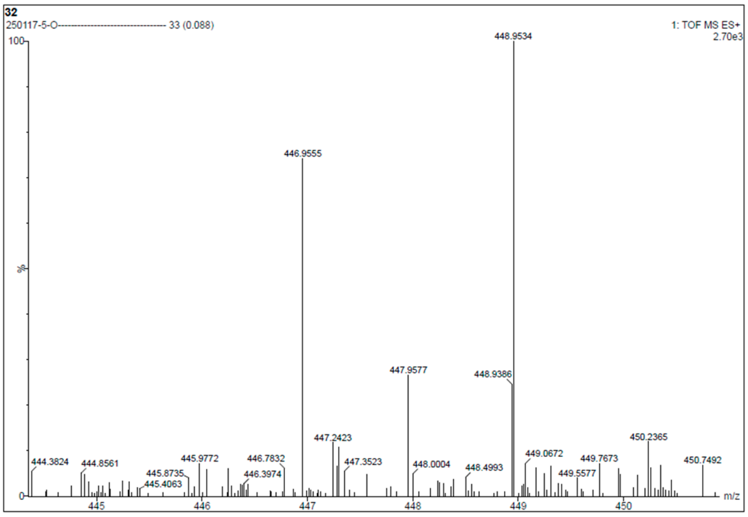

The ^{1}H NMR spectrum of compound **5p**

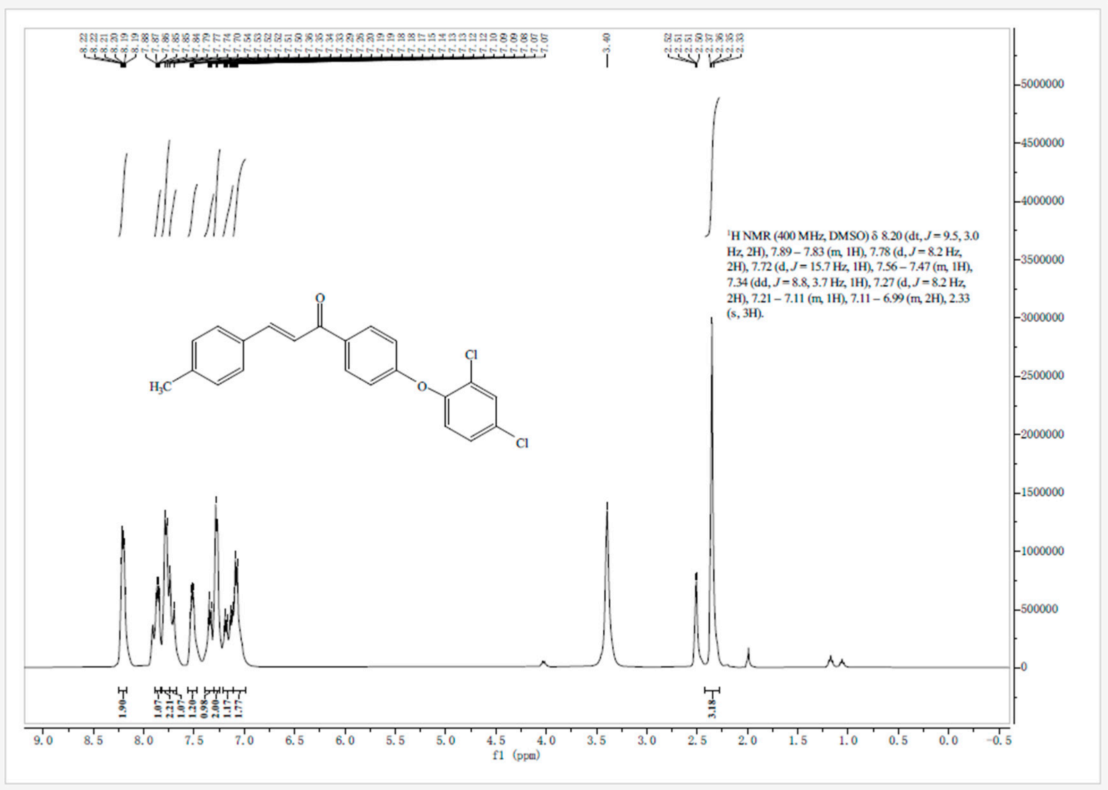

The ^{13} C NMR spectrum of compound **5p**

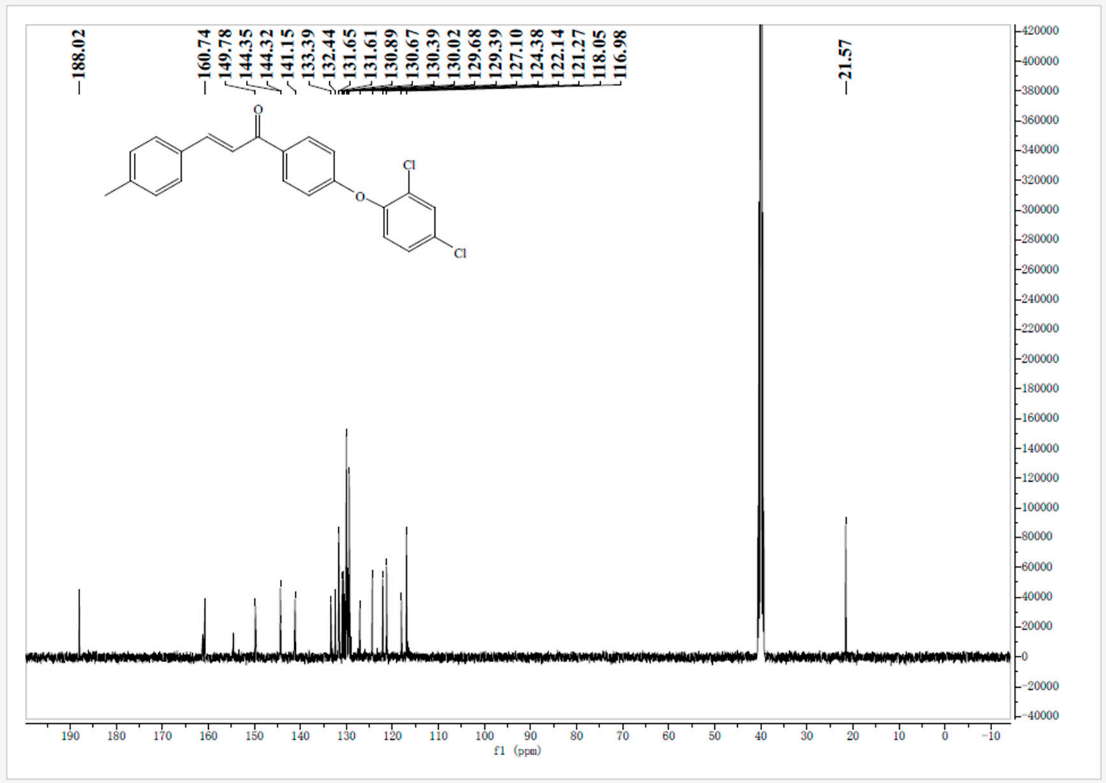

The HRMS of compound **5p**

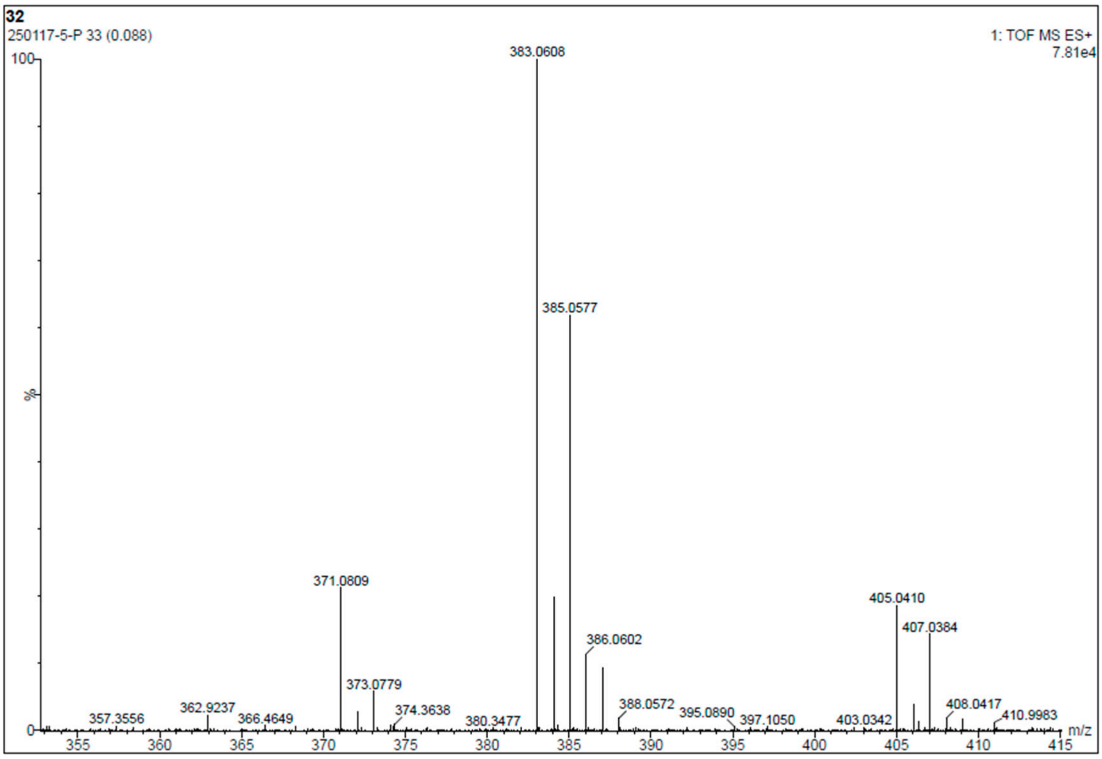

The IR of compound **5p**

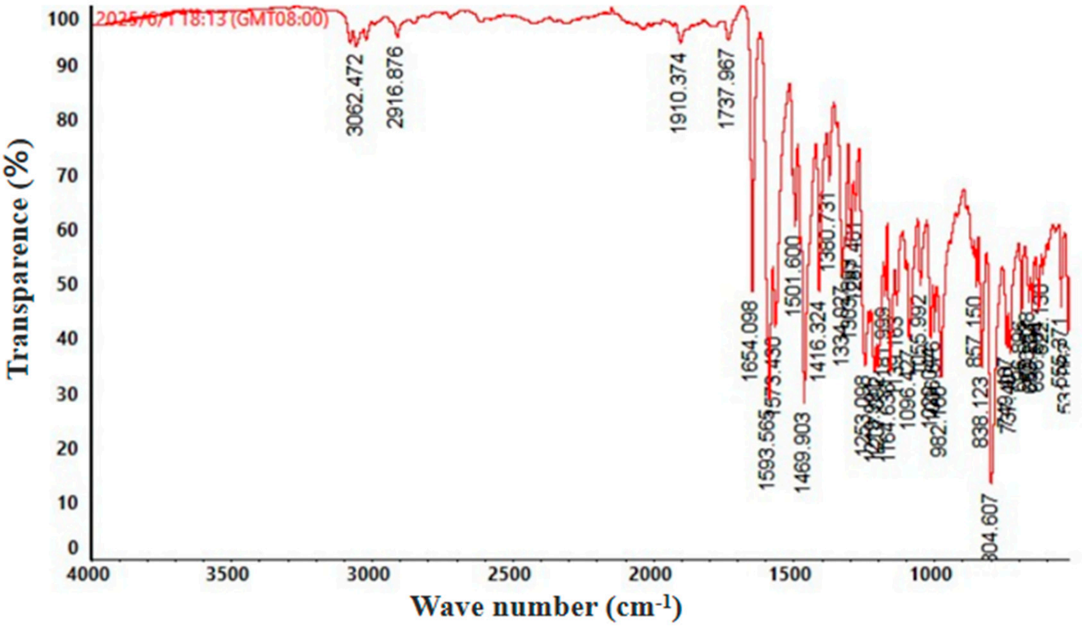

The ^{1}H NMR spectrum of compound **5q**

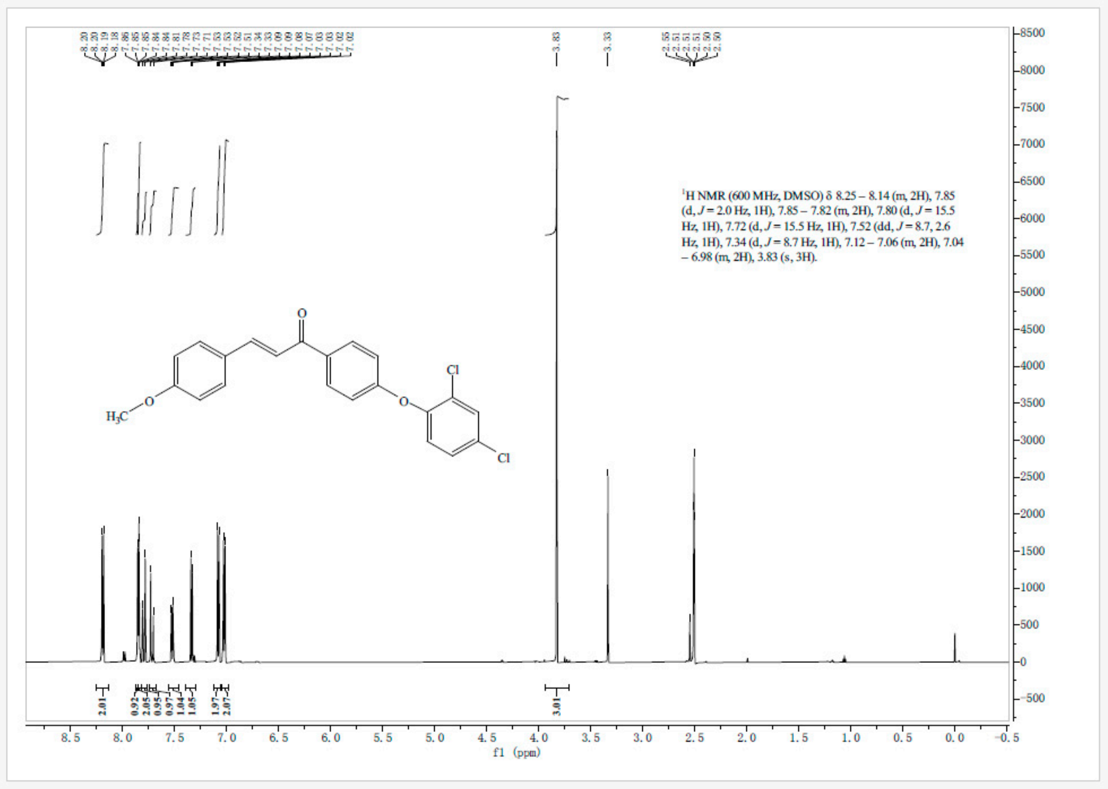

The ^{13} C NMR spectrum of compound **5q**

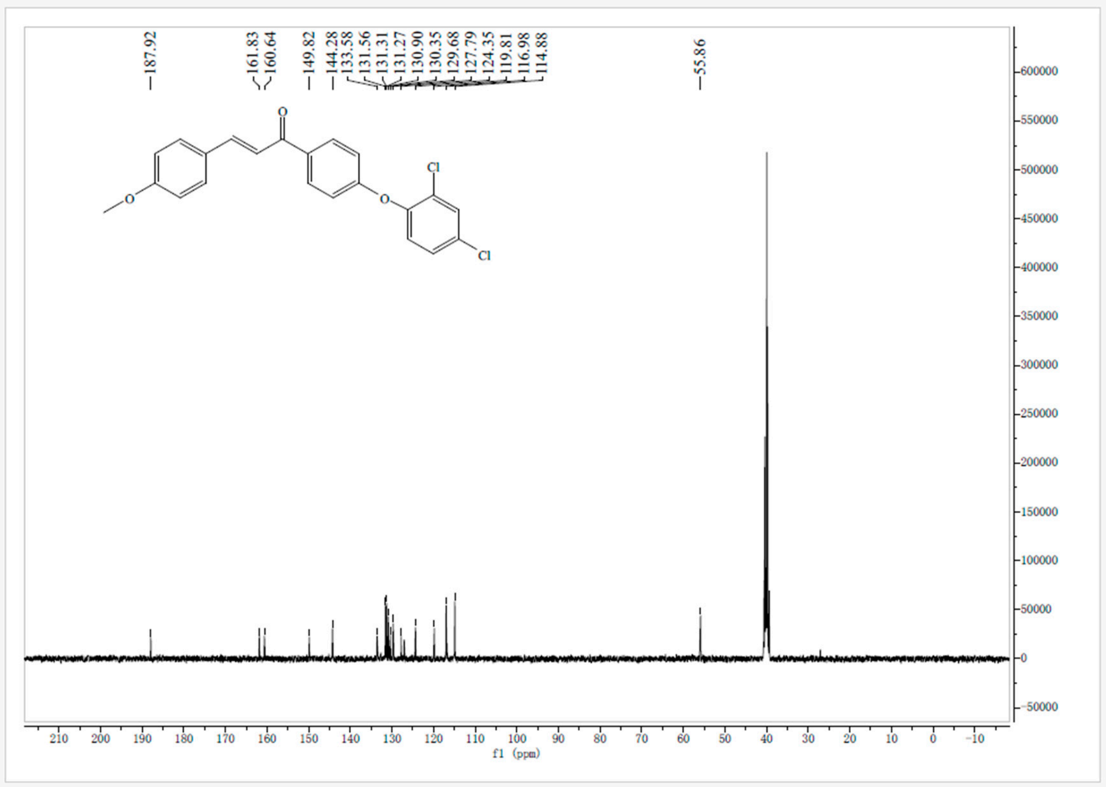

The HRMS of compound **5q**

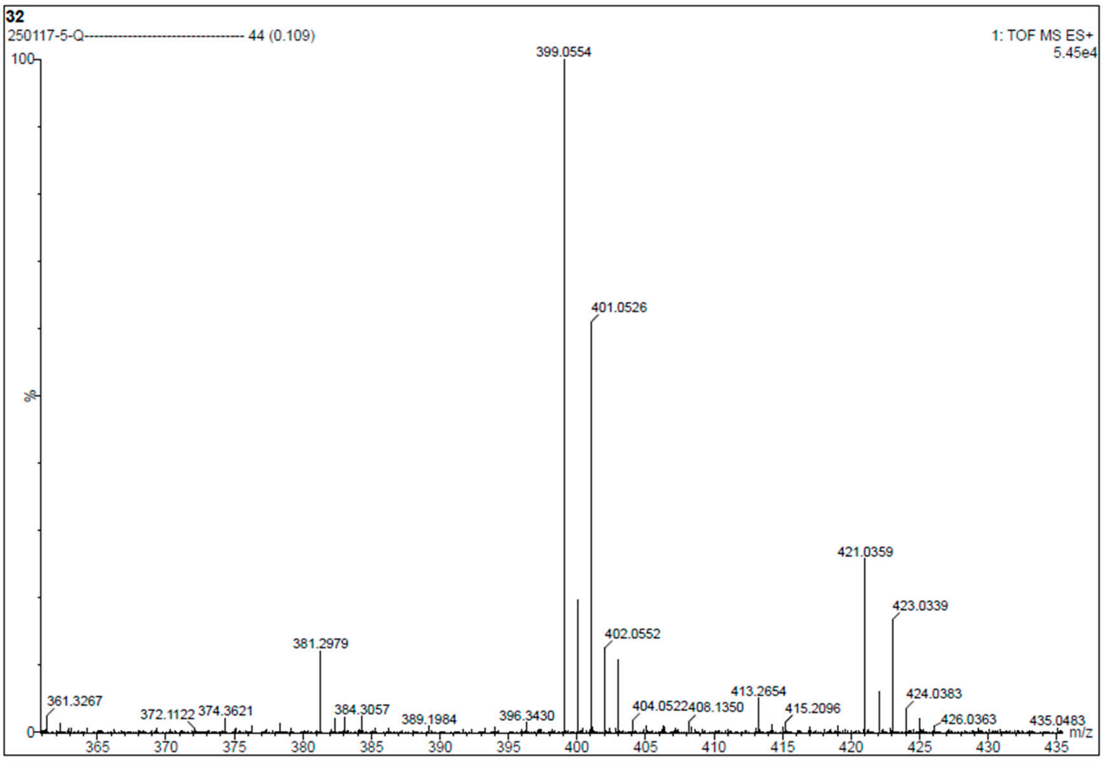

The IR of compound **5q**

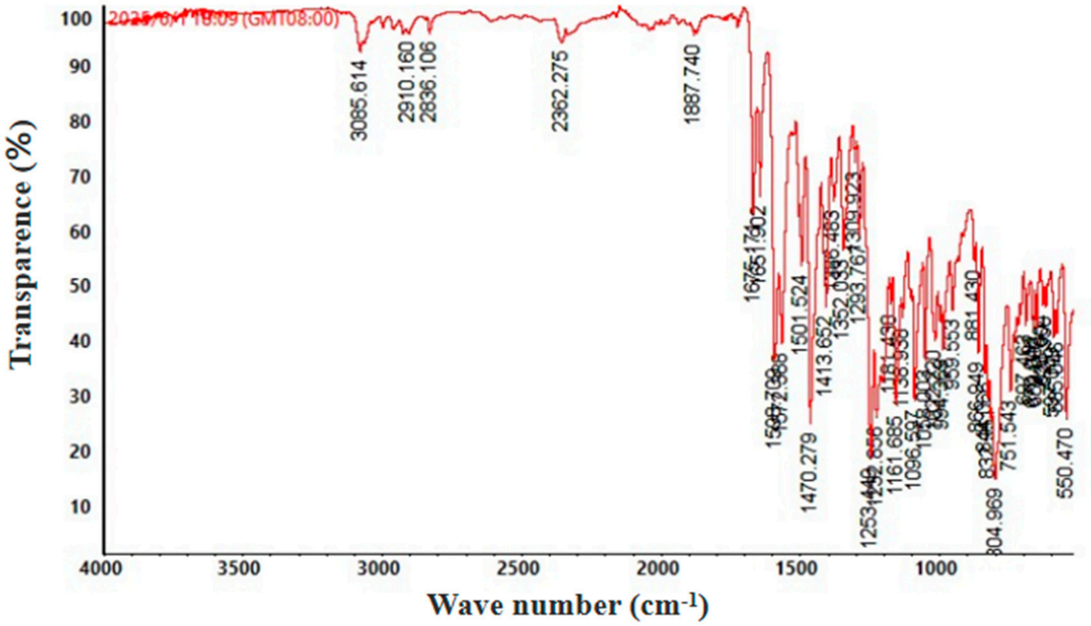

The ^{1}H NMR spectrum of compound **5r**

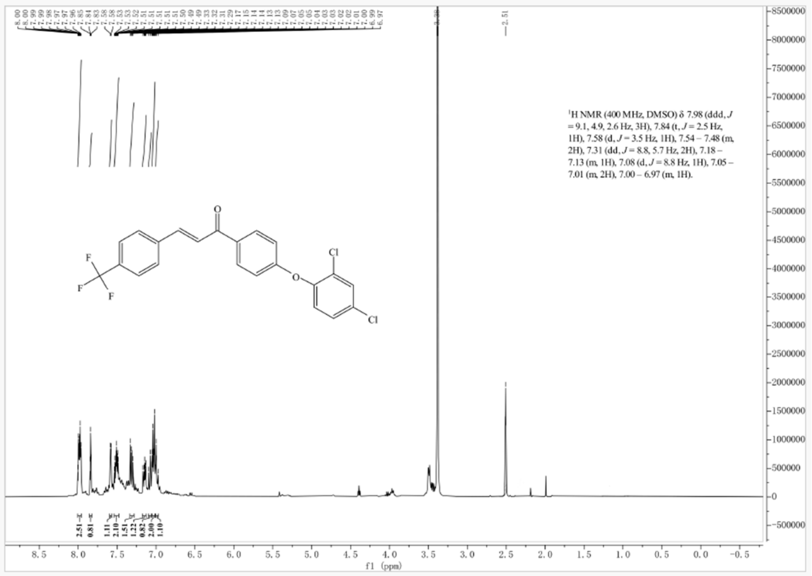

The HRMS of compound **5r**

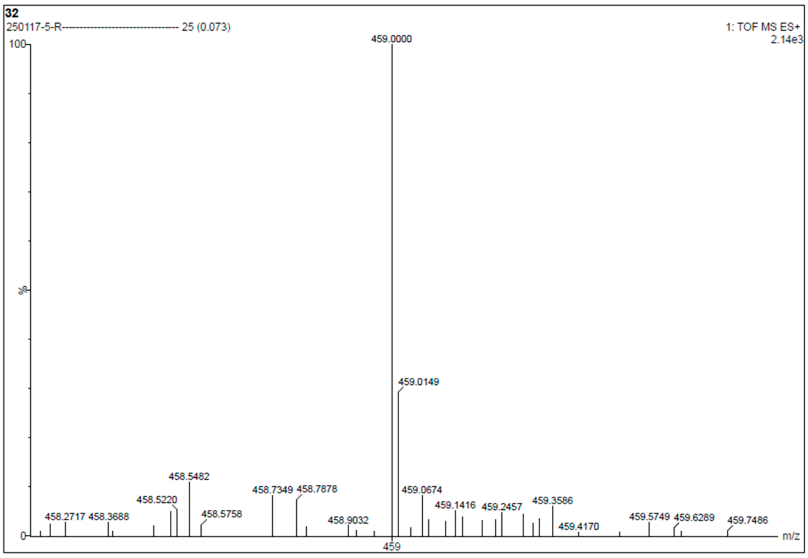

The IR of compound **5r**

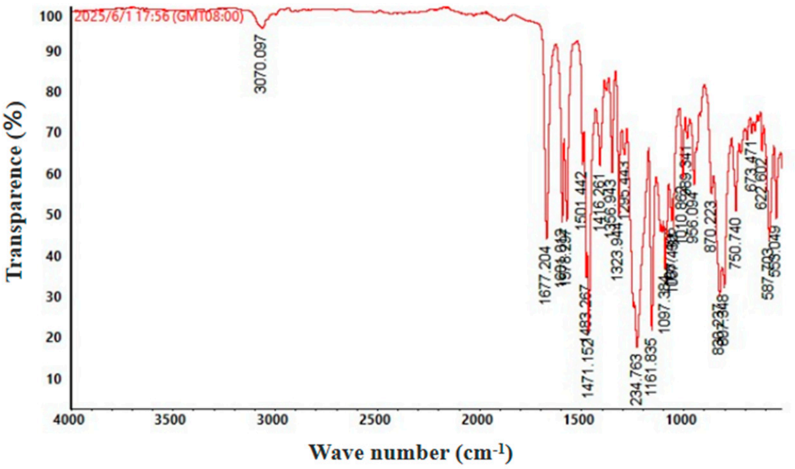

The ^{1}H NMR spectrum of compound **5s**

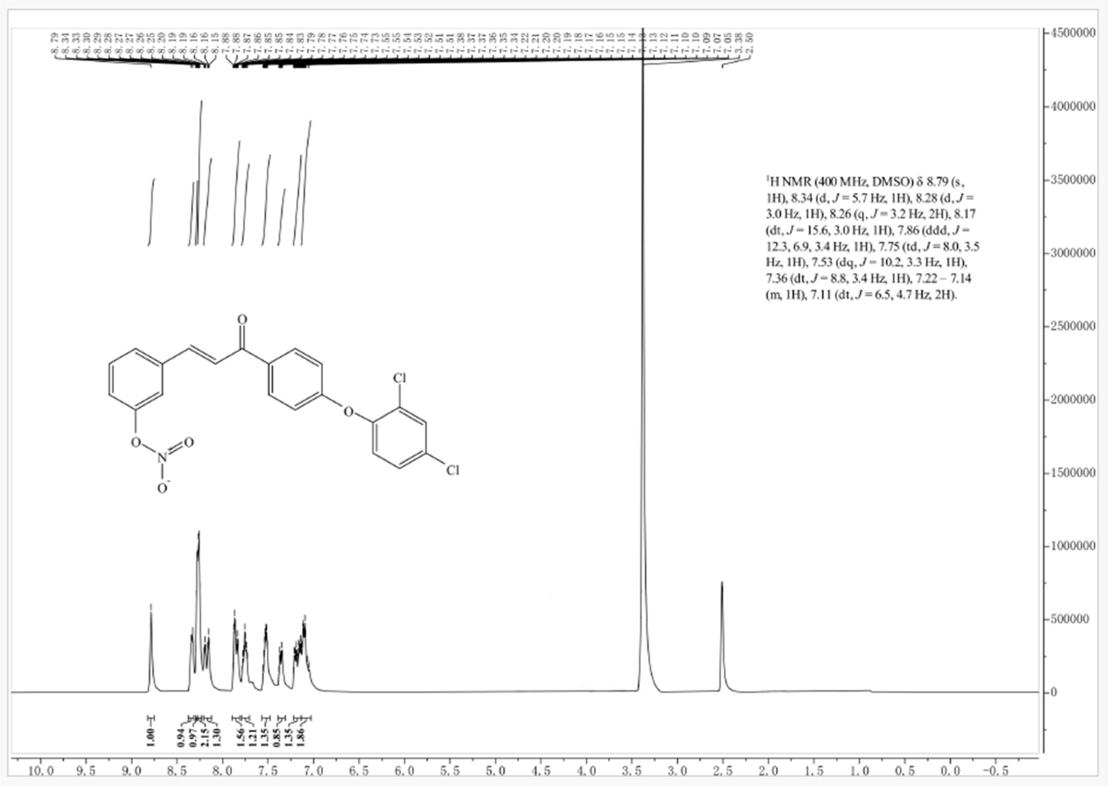

The ^{13} C NMR spectrum of compound **5s**

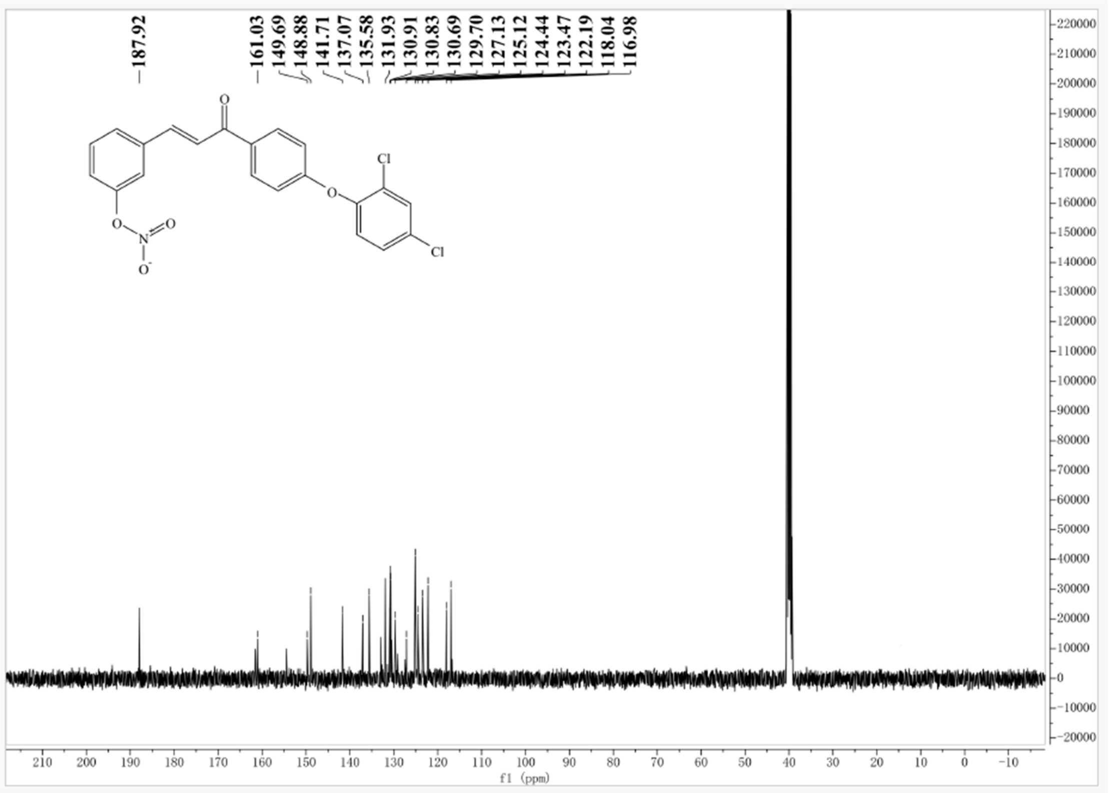

The HRMS of compound **5s**

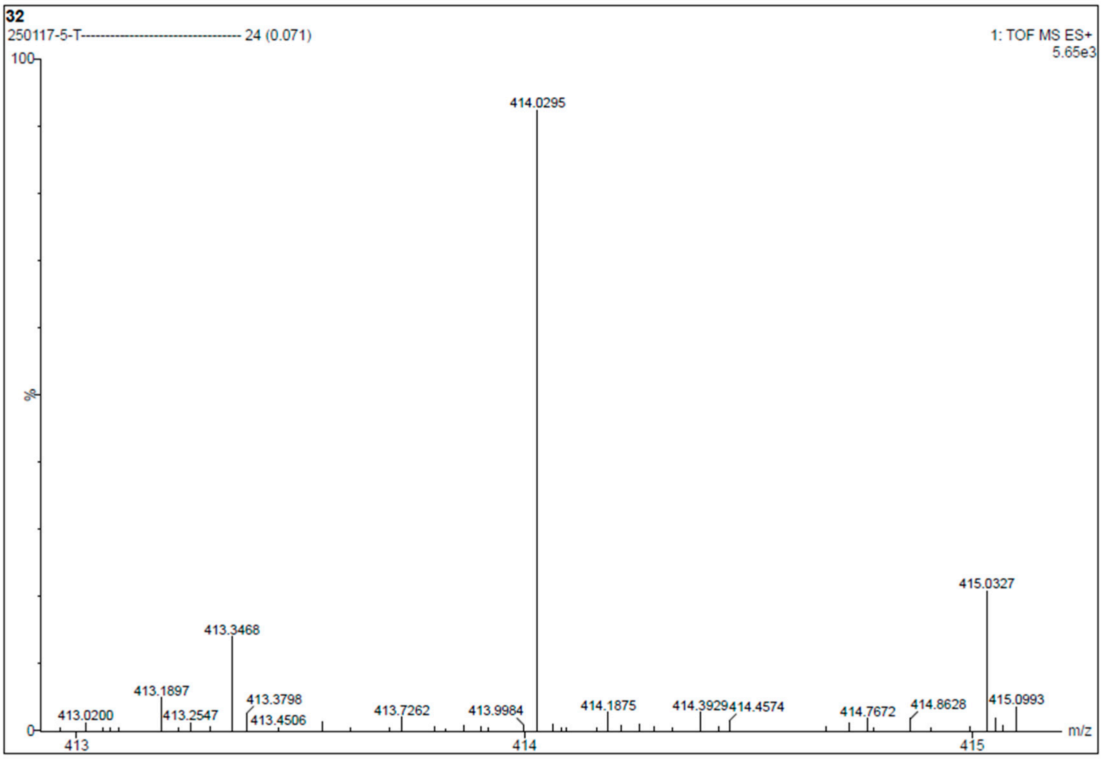

The IR of compound **5s**

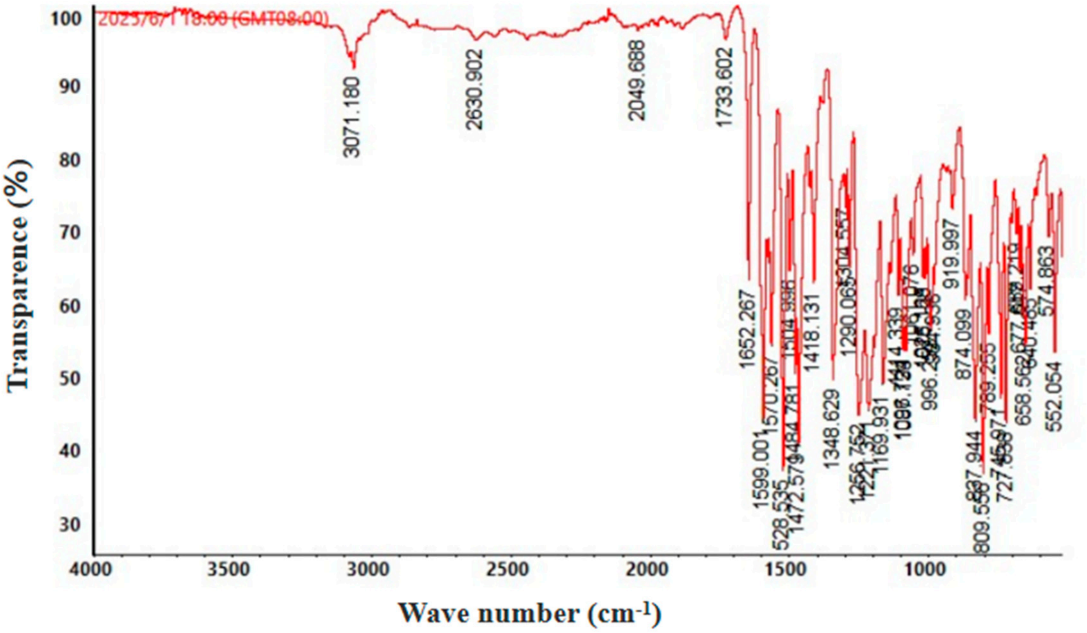

The ^{1}H NMR spectrum of compound **5t**

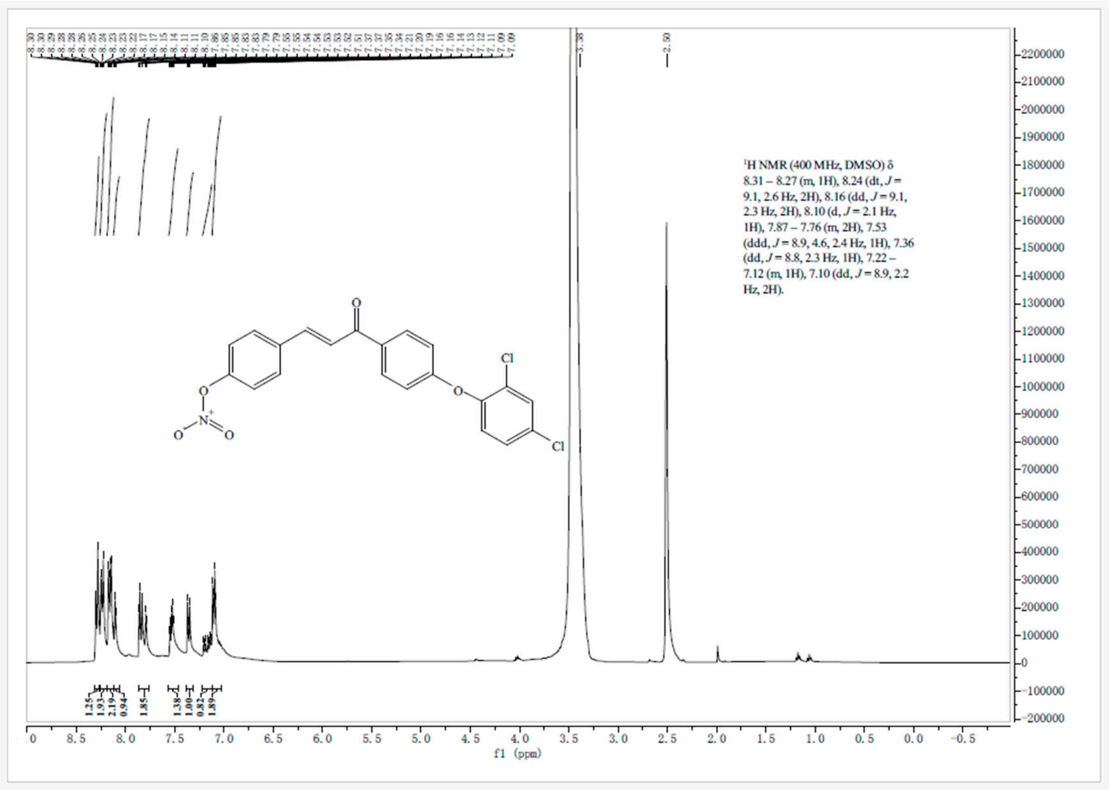

The ^{13} C NMR spectrum of compound **5t**

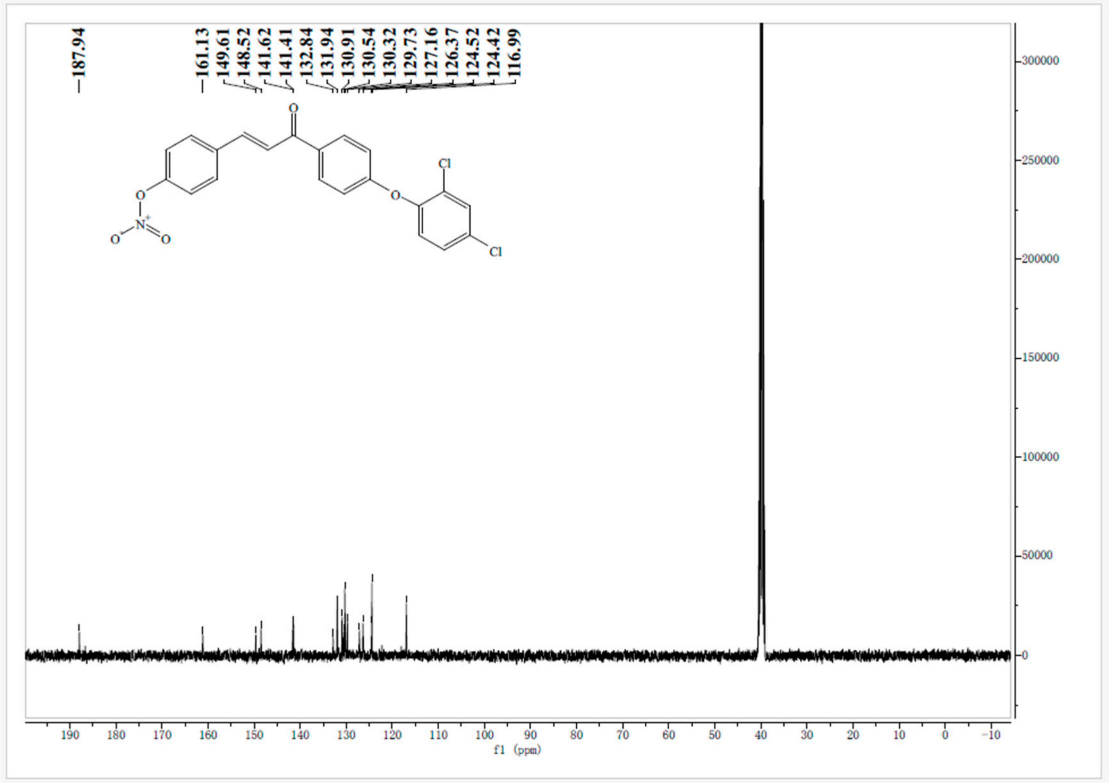

The HRMS of compound **5t**

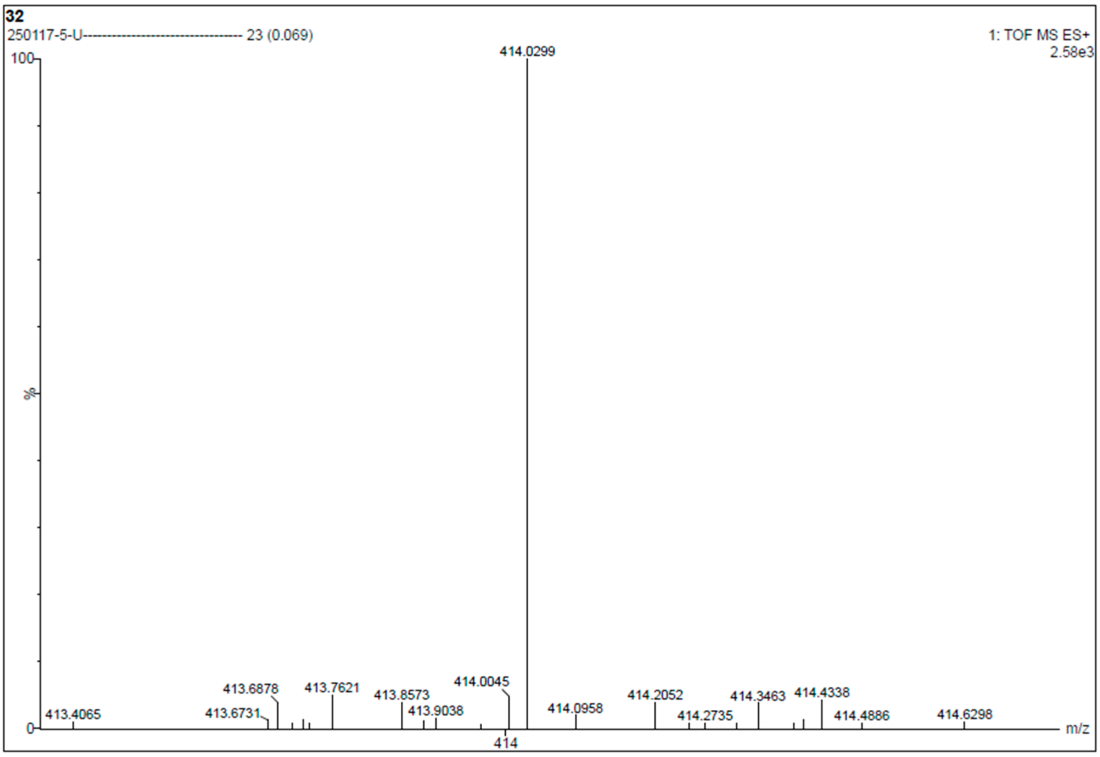

The IR of compound **5t**

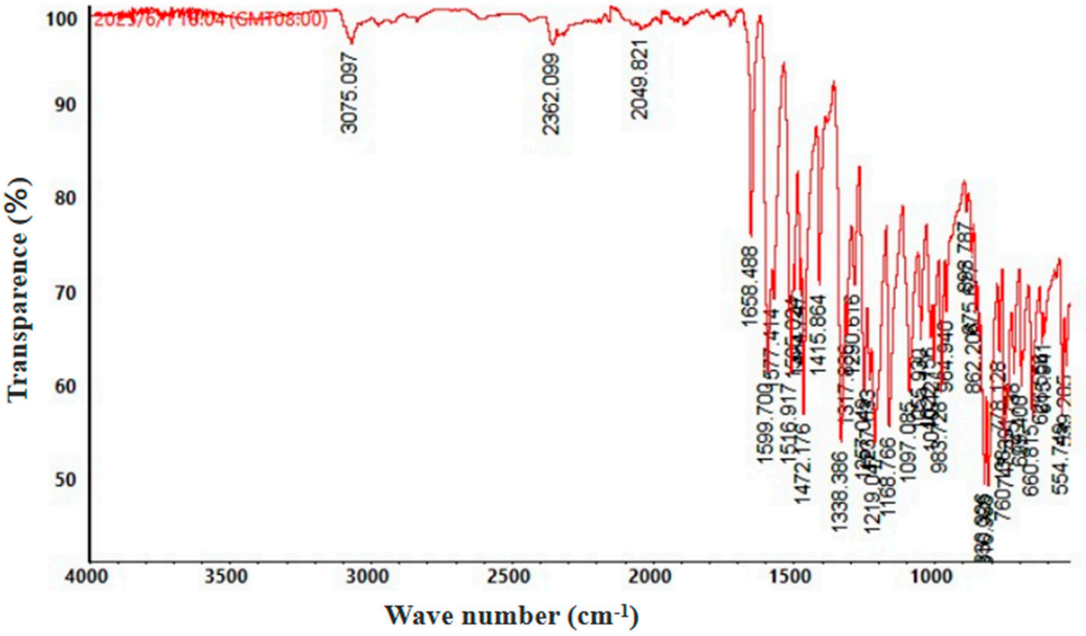

The ^{1}H NMR spectrum of compound **5u**

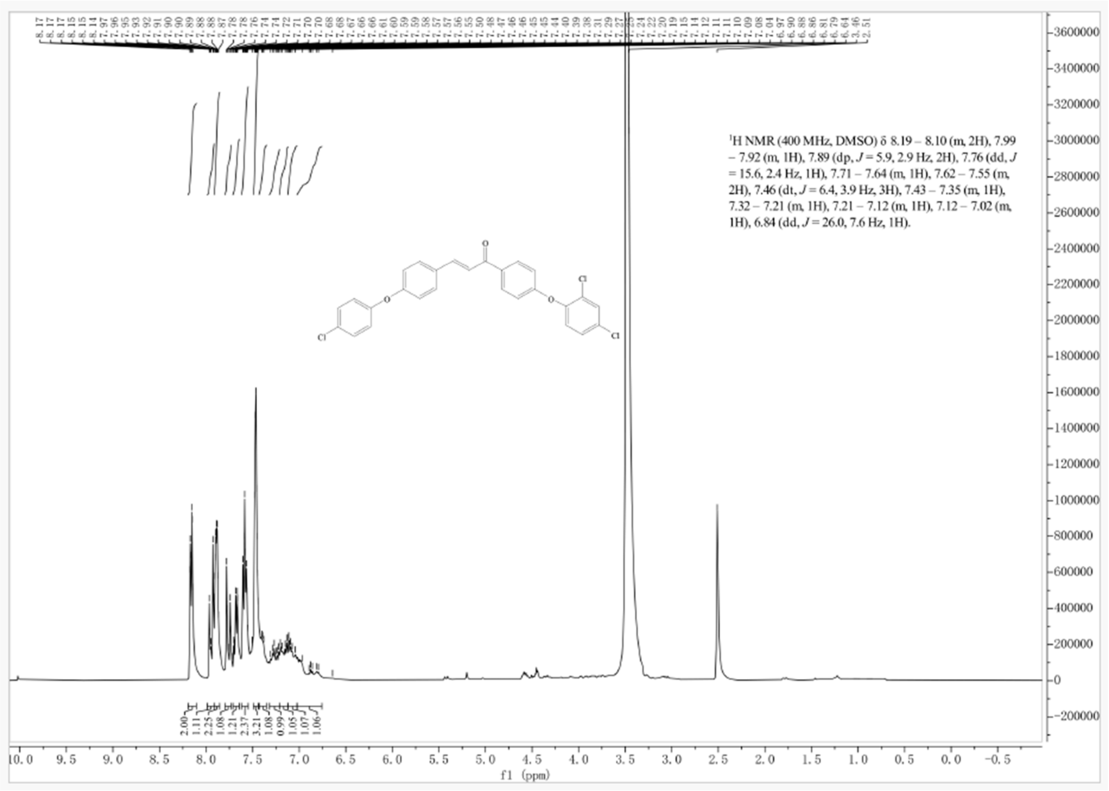

The [13] C NMR spectrum of compound **5u**

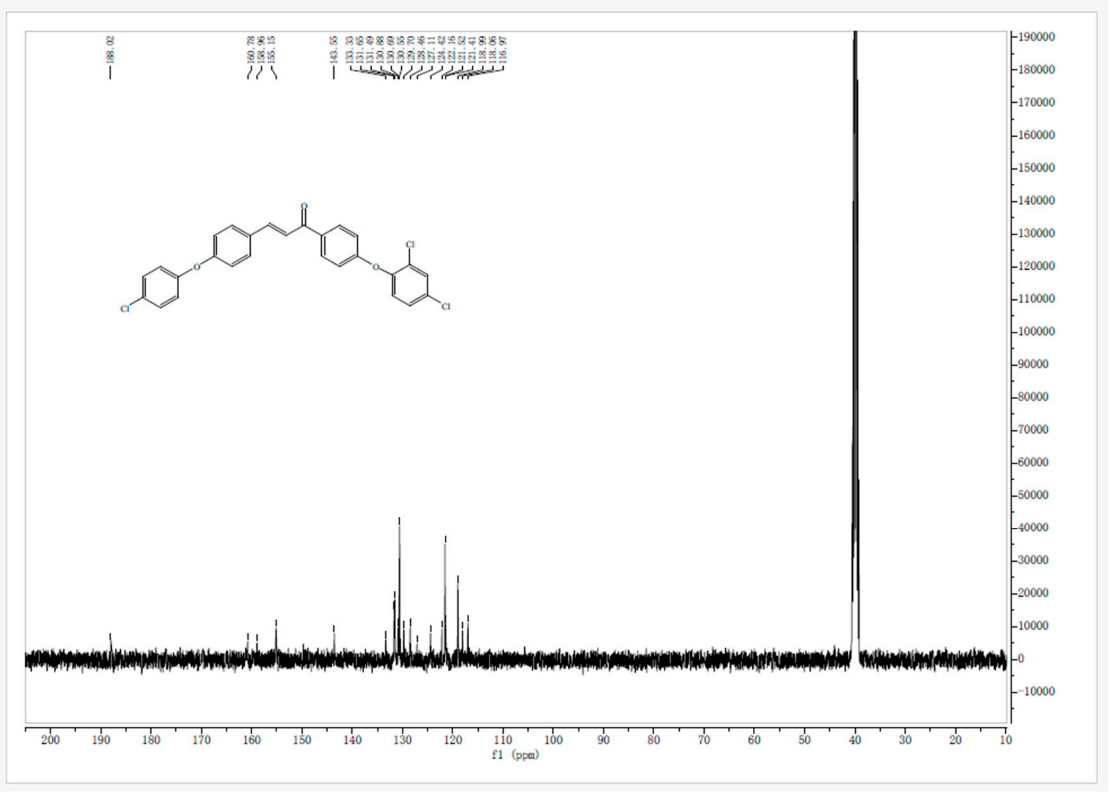

The HRMS of compound **5u**

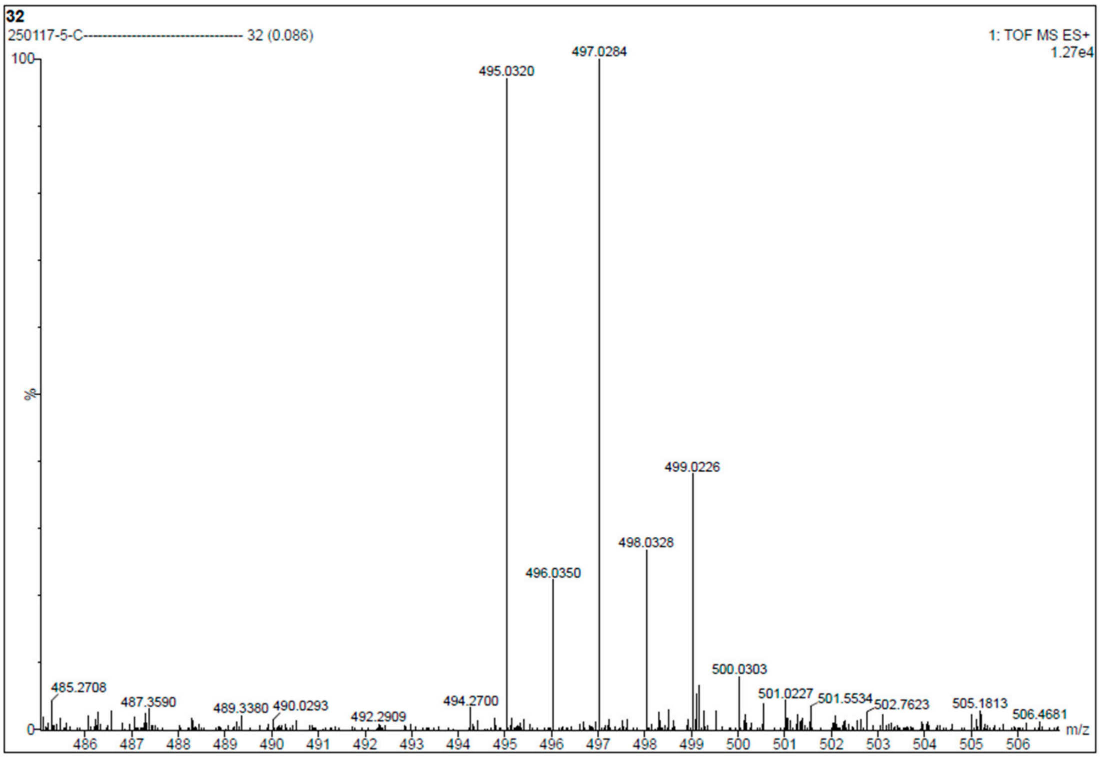

The IR of compound **5u**

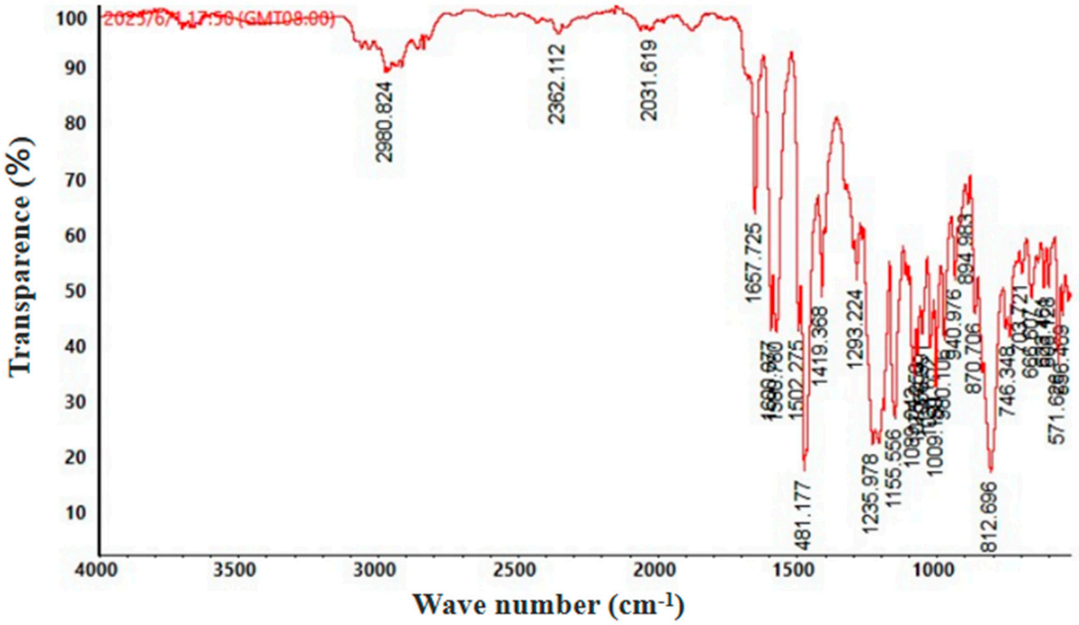

Supplement: Supplementary file 1 [file molecules-30-02575-s001.zip › molecules-3670334-supplementary.pdf]
